# Unveiling Alternative Oxidation Pathways and Antioxidant and Cardioprotective Potential of Amaranthin-Type Betacyanins from Spinach-like *Atriplex hortensis* var. ‘*Rubra*’

**DOI:** 10.1021/acs.jafc.3c03044

**Published:** 2023-10-04

**Authors:** Agnieszka Kumorkiewicz-Jamro, Renata Górska, Małgorzata Krok-Borkowicz, Przemysław Mielczarek, Łukasz Popenda, Kateryna Lystvan, Elżbieta Pamuła, Sławomir Wybraniec

**Affiliations:** † Department of Chemical Technology and Environmental Analysis, Faculty of Chemical Engineering and Technology, Cracow University of Technology, Warszawska 24, 31-155 Cracow, Poland; ‡ South Australian Health and Medical Research Institute, Adelaide 5000, SA, Australia; § Faculty of Health and Medical Sciences, University of Adelaide, Adelaide 5000, SA, Australia; ∥ Department of Biomaterials and Composites, Faculty of Materials Science and Ceramics, AGH University of Science and Technology, Al. Mickiewicza 30, 30-059 Cracow, Poland; ⊥ Department of Analytical Chemistry and Biochemistry, Faculty of Materials Science and Ceramics, AGH University of Science and Technology, Al. Mickiewicza 30, 30059 Cracow, Poland; # Laboratory of Proteomics and Mass Spectrometry, Maj Institute of Pharmacology, Polish Academy of Sciences, Smętna 12, 31-343 Cracow, Poland; ∇ NanoBioMedical Centre, Adam Mickiewicz University, Wszechnicy Piastowskiej 3, 61-614 Poznań, Poland; ○ Department of Genetic Engineering, Institute of Cell Biology and Genetic Engineering of National Academy of Sciences of Ukraine (NASU), Academika Zabolotnoho, 148, 03143 Kyiv, Ukraine

**Keywords:** betalains, oxidation, Atriplex hortensis var. *rubra*, antioxidant activity, cardioprotective activity

## Abstract

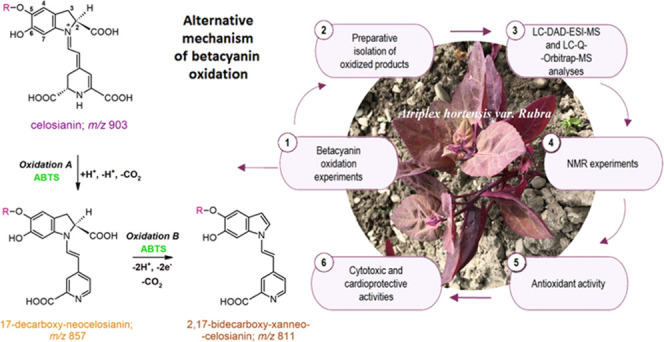

A comprehensive oxidation mechanism was investigated for amaranthin-type betacyanins with a specific glucuronosylglucosyl moiety isolated from *Atriplex hortensis* '*rubra'* using liquid chromatography coupled to diode array detection and electrospray ionization tandem mass spectrometry (LC-DAD-ESI-MS/MS) and LC-Quadrupole-Orbitrap-MS (LC-Q-Orbitrap-MS). By employing one-dimensional (1D) and two-dimensional (2D) NMR, this study elucidates the chemical structures of 2,2′-azino-bis(3-ethylbenzothiazoline-6-sulfonic acid) diammonium salt (ABTS)-oxidized celosianins for the first time. These findings demonstrate alternative oxidation pathways for acylated betacyanins compared to well-known betanidin, betanin, and gomphrenin pigments. Contrary to previous research, we uncover the existence of 17-decarboxy-neo- and 2,17-bidecarboxy-xanneo-derivatives as the initial oxidation products without the expected 2-decarboxy-xan forms. These oxidized compounds demonstrated potent free radical scavenging properties. Celosianin (IC_50_ = 23 μg/mL) displayed slightly higher antioxidant activity compared to oxidized forms, 17-decarboxy-neocelosianin (IC_50_ = 34 μg/mL) and 2,17-bidecarboxy-xanneocelosianin (IC_50_ = 29 μg/mL). The oxidized compounds showed no cytotoxic effects on H9c2 rat cardiomyoblasts (0.1–100 μg/mL). Additionally, treatment of H9c2 cells with the oxidized compounds (0.1–10 μg/mL) elevated glutathione levels and exhibited protective effects against H_2_O_2_-induced cell death. These findings have significant implications for understanding the impact of oxidation processes on the structures and biological activities of acylated betalains, providing valuable insights for future studies of the bioavailability and biological mechanism of their action *in vivo*.

## 1 Introduction

Betalains are a family of natural plant-derived pigments comprised of red-violet betacyanins and yellow-orange betaxanthins.^1^ Currently, betalains have been found in the spinach-type *Atriplex hortensis* var. *rubra*, including amaranthin-type pigments, such as amaranthin, celosianin, and argentianin. *A. hortensis'rubra'* is a promising novel source of nutrients, resilient to harsh environmental and growing conditions, providing an alternative to the widely studied beetroot.^2,3^ It is well established that betalains are beneficial to human health, exhibiting chemopreventive, antioxidant, and anti-inflammatory properties.^4^ In our previous report, we demonstrated that *A. hortensis* '*rubra'* extracts, along with isolated amaranthin and celosianin, also possess antioxidant and cardioprotective properties.^2^

Currently, there is a demand in the food industry for natural additives with nutritional value, including plant pigments.^1^ Betalains and betalain-rich plant extracts are recognized as safe alternatives to synthetic food colorants, enhancing redness and extending the shelf life of food products.^5−7^ However, the application of natural dyes is constrained by their high susceptibility to degradation under unfavorable conditions during food processing, leading to color loss and a decline in taste quality.^8^ It is known that betalains undergo transformation during food preparation^9,10^ and digestion.^11,12^ However, research on the composition of degradation products, their functions, and potential toxicity is limited.

The studies conducted by Sawicki et al.^11,12^ reported the presence of native betalains as well as decarboxylated, deglucosylated, and dehydrogenated derivatives in plasma and urine after the consumption of red beetroot products. Furthermore, the study suggests that the dosage of betacyanins affects the degradation rate in gastric content as well as the absorption, metabolism, and excretion of these pigments. So far, only a limited number of studies have explored betalain derivatives arising from hydrolysis,^13^ oxidation,^14−16^ conjugation,^17,18^ and heating^19,20^ processes, which occur during food processing and pigment extraction. A promising potential in incorporating betalains and their derivatives into intelligent food packaging^21^ and edible, biodegradable biopolymer-based films is currently being explored.^22^ This innovative approach allows for real-time detection of food spoilage and freshness based on the color change resulting from the generation of betalain derivatives.^22^ Further research is also needed to investigate their stability,^23^ impact on human health,^24^ and potential applications.

Furthermore, research on the oxidation of betalains is crucial to understanding their mechanism of action, stability, properties, and potential as innovative derivatives for betalain-based products. The reactions that occur during oxidation can vary depending on the chemical structures and the presence of different functional groups in betalains.^25^

In this study, we identify oxidized derivatives of amaranthin, argentianin, and celosianin isolated from *A. hortensis* var. *rubra*. The nonenzymatic oxidation mechanism of betalains was investigated by using liquid chromatography coupled to diode array detection and electrospray ionization tandem mass spectrometry (LC-DAD-ESI-MS/MS), LC-Quadrupole-Orbitrap-MS (LC-Q-Orbitrap-MS), and NMR techniques. For the first time, we successfully isolated and purified oxidized forms of celosianin using ion-exchange chromatography and preparative high-performance liquid chromatography (HPLC) while also determining their chemical structures through MS fragmentation data and NMR analyses. Furthermore, the antioxidant activities of celosianin and its oxidized derivatives were evaluated by 2,2′-azino-bis(3-ethylbenzothiazoline-6-sulfonic acid) diammonium salt (ABTS), ferric reducing antioxidant power (FRAP), and electrochemical methods. Preliminary studies have also been conducted to assess the cardioprotective effect of oxidized celosianins to determine whether betalains can alleviate drug-resistance and cardiotoxicity. Our findings indicate that the chemical structures of amaranthin-type betacyanins, specifically those with a glucuronosylglucosyl moiety, influence their oxidation pathways and the resulting oxidized products. This sheds light on the alternative mechanisms of their transformations during food processing and digestion. The results may help in interpreting the profiles of acylated betalains and their derivatives *in vivo*, as well as their physiological functions upon consumption.

## 2 Materials and Methods

### 2.1 Reagents

Reagents of HPLC and MS grade, including water, formic acid, methanol, acetone, and ethanol, as well as reagents for antioxidant activity assays, such as 6-hydroxy-2,5,7,8-tetramethylchroman-2-carboxylic acid (Trolox), 2,2′-azobis(2-amidinopropane)-dihydrochloride (AAPH), sodium fluorescein, 2,3,5-triphenyltetrazolium chloride (TPTZ), ferric chloride hexahydrate, sodium acetate trihydrate, 2,2′-azino-bis(3-ethylbenzothiazoline-6-sulfonic acid) diammonium salt (ABTS), and gallic acid, were purchased from Sigma-Aldrich (St. Louis, MO).

Reference betacyanins, including amaranthin, argentianin, and celosianin, along with their respective isoforms, have been previously isolated from *Amaranthus cruentus* L., *A. hortensis* var. *rubra*, and *Celosia plumosa* L., and their chemical structures have been fully elucidated.^2,26,27^

### 2.2 Plant Material

*A. hortensis* var. *rubra* was sown in May in a sunny location with well-drained soil and harvested in September 2020 at the Botanical Garden of Jagiellonian University, Institute of Botany in Cracow.

### 2.3 Extraction and Isolation of Betacyanins from *A. hortensis* var. *Rubra*

Betacyanins for oxidation experiments were extracted from *A. hortensis* var. *rubra* plant using 20% acetone acidified with 1% formic acid (*v/v*) through maceration. A 30 g of extract was obtained from 1 kg of plant material by repeating the extraction process three times. The extract was then purified using a weak anion exchanger (Sepra ZT-WAX 30 μm polymer, 85 Å, Phenomenex, Torrance) and semipreparative liquid chromatography, following previous reports.^2,3^

### 2.4 Betacyanin Oxidation by ABTS Cation Radical

The oxidation experiments for isolated and purified amaranthin, argentianin, and celosianin were conducted by combining 30 μL of each pigment (200 μM) with 40 μL of 1.2 mM ABTS cation radical, as previously described.^17^ The reactions were carried out in the presence of 20 μL of acetate (pH 3–5.5) and phosphate (pH 6–7.4) buffers (25 mM). Transparent 96-well plates of a Microplate Reader Infinite 200 (Tecan Austria GmbH, Grödig/Salzburg, Austria) were used, and the reactions were performed at 20 °C. The total volume of each well was adjusted to 200 μL by adding water to the reaction mixture. Spectra were collected over 30 min by spectrophotometric detection in the wavelength range of 350–600 nm. After incubation for 5, 20, 40, 60, and 80 min, the reaction mixtures were analyzed by LC-MS/MS through direct injection of 20 μL into the system.

Celosianin oxidation was also carried out on a semipreparative scale by combining 50 mL of twice purified pigment (1 mM) with 50 mL of 1.3 mM ABTS cation radical in the presence of 15 mL of acetate buffer solution (0.2 M) and 110 mL of deionized water. The progression of the reaction was monitored by LC-MS. After 80 min, the reaction was terminated by removing ABTS from using a strong anion exchanger (Sepra ZT-SAX 30 μm polymer, 85 Å, Phenomenex, Torrance). The fraction containing the pigments was eluted from the column using 2% aqueous formic acid in a 50% acetone eluent. The resulting eluate was evaporated and subjected to isolation and purification using semipreparative HPLC.

### 2.5 Isolation and Purification of Celosianin and Its Oxidized Products by Semipreparative Chromatography

The extract of *A. hortensis* var. *rubra*, initially purified on WAX, along with the oxidation products, underwent additional purification on prep-HPLC. The isolation of amaranthin, argentianin, celosianin, and oxidized celosianin derivatives was performed using a Shimadzu LC-20AD preparative chromatographic system (Kyoto, Japan) following a previously established procedure.^2,16^

The purification of the oxidized celosianin derivatives was carried out using a gradient system composed of 0.5% aqueous formic acid (solvent A) and acetone (solvent B) as follows: 0 min, 0% B; linear increase to 7% B at 5 min; linear increase to 8% B at 15 min; linear increase to 10% B at 25 min; linear increase to 12% B at 30 min; and linear increase to 70% B at 35 min. The injection volume was 40 mL, and the flow rate was 50 mL/min. Detection was performed by using a PDA UV/Vis detector at 540, 505, 460, and 420 nm. The eluates were pooled, concentrated under a reduced pressure at 25 °C, and subsequently freeze-dried. The separation was performed on a reverse-phase column (250 mm × 30 mm, 10 μm C18(2) Luna, Phenomenex, Torrance, CA).

Further purification of the oxidized pigments was carried out on a reverse-phase Phenomenex column, 250 mm × 10 mm, 10 μm (Phenomenex, Torrance, CA), using the following gradient system composed of 0.05% aqueous formic acid (solvent A) and acetone (solvent B) as follows: 0 min, 0% B; linear increase to 10% B at 5 min; linear increase to 12% B at 15 min; linear increase to 14% B at 25 min; and linear increase to 70% B at 30 min. The injection volume was 20 mL, and the flow rate was 40 mL/min. Detection was performed at characteristic wavelengths for oxidized betacyanins using a PDA detector at 480, 440, and 420 nm. The obtained fractions were concentrated under reduced pressure at 20 °C, freeze-dried, and stored at −20 °C for further research.

### 2.6 LC-DAD-ESI-MS/MS and LC-Q-Orbitrap-MS Analyses

At each stage of the research, qualitative analyses of the samples were conducted using high-performance liquid chromatography coupled with low-resolution mass spectrometry with electrospray ionization, utilizing the LC-MS-8030 system controlled by LabSolutions software version 5.91 SP1 (Shimadzu, Japan). Chromatographic separation was conducted on a 150 mm × 4.6 mm, 5.0 μm Kinetex C_18_ column (Phenomenex, Torrance, CA) protected by a guard column filled with the same stationary phase material (Phenomenex). The mobile phase consisted of 2% aqueous formic acid (solvent A) and methanol (solvent B). The binary gradient elution for betacyanins and their oxidized forms was as follows: 0 min, 10% B; linear increase to 40% B at 12 min, 40% B; and linear increase to 80% B at 15 min. The total run time was 19 min. The column was maintained at 40 °C, and the flow rate was set at 0.5 mL/min.

Data were recorded in positive ion polarity using selected ion monitoring (SIM) and scan mode with *m*/*z* ranging from 100 to 2000 Da, as well as in product ion scan mode (PIS) for fragmentation experiments. The following parameters were set for the analyses: an electrospray voltage of 4.5 kV, a capillary temperature of 250 °C, a nebulizing gas flow rate of 1.5 L/min, and a curved desolvation line (CDL) and heat block temperature of 230 °C. Nitrogen was used as a gas for the stray, and argon was used as the collision gas for the collision-induced dissociation (CID) experiments. The relative collision energies for the MS/MS analyses were set to −35 V.

The LC-high-resolution MS (LC-HRMS) data acquisition and analyses were performed using the Orbitrap Exploris 240 Mass Spectrometer with Chromeleon 7.2.10 and Xcalibur 4.3 software (Thermo Fisher Scientific). The HRMS data were acquired in the *m*/*z* range of 120–1200 with a full width at half-maximum resolution 120,000 (fwhm) at *m*/*z* 200 and a standard automatic gain control (AGC) target value in the full-scan mode. The maximum isolation time was set to auto mode. The acquisition mode used was the product ion scan mode, in which targeted precursors were isolated and fragmented in the higher-energy collision-induced dissociation (HCD) cell. The selected precursor ions were fragmented in a higher-energy collision-induced dissociation (HCD) cell, and the fragmentation ions (MS^2^) were analyzed in the Orbitrap analyzer. For the MS^2^ experiments, the fragment ions of the target pigments were collected in the HCD mode, with 5 scans and dynamic exclusion (threshold intensity 5000). The collision energy was normalized for small molecules, and the isolation window was set to an *m*/*z* of 1.5. The resolution was 30,000, and the AGC target value was standard. The *m*/*z* range was set to auto mode, and the maximum isolation time was 54 ms. The number of microscans per MS/MS scan was 1.

### 2.7 NMR Experiments

NMR analyses of 2,17-bidecarboxy-xanneocelosianin 20 and 2-decarboxy-xanneocelosianin 22 were performed on an Agilent DD2 800 (18.8 T) spectrometer (Agilent Technologies, Santa Clara, CA) in DMSO-*d*_6_/TFA-*d*. Analysis of 17-decarboxy-neocelosianin was performed in D_2_O on a Bruker Avance III 700 (16.4 T) spectrometer (Bruker Co., Billerica, MA) using a QCI CryoProbe at 295 K. Solvent suppression was achieved by using low-power presaturation pulses during the relaxation delay. The one-dimensional (1D) (^1^H, ^13^C) and two-dimensional (2D) (COSY, HSQC, HMBC, TOCSY, and NOESY (gradient enhanced)) experiments were performed by using standard Agilent or Bruker pulse sequences and acquisition parameters. Chemical shifts were determined relative to the internal 3-(trimethylsilyl)-2,2,3,3-tetradeuteropropionic acid (TMSP-*d*_4_) (δ_H_ = 0.00 ppm, δ_C_ = 0.0 ppm) for 17-decarboxy-neocelosianin 18 or the residual DMSO-*d*_6_ peak (δ_H_ = 2.50 ppm, δ_C_ = 39.5 ppm) for the other pigments.

### 2.8 Electrochemical Measurements

All voltammetric measurements were performed using the electrochemical analyzer M161 (model EA9/M151E) connected to the electrode stand M164 (both MTM-ANKO, Poland) with EALab 2.1 software. The standard three-electrode system consisted of a bare glassy carbon electrode (GCE, φ = 1.8 mm, BASi) as the working electrode, a silver chloride electrode (Ag/AgCl, 3 M KCl, Mineral, Poland) as the reference electrode, and a platinum wire as the auxiliary electrode. The electrochemical activity of amaranthin, argentianin, and celosianin was elucidated using cyclic voltammetry (CV) and differential pulse voltammetry (DPV) techniques. CV voltammograms were recorded at scan rates ranging from 25 to 1000 mV/s at 25 °C in 0.1 M acetate (pH 3–5) and phosphate (6–7) buffer solutions. Prior to each experiment, 1 mL of a 1.4 mM pigment aqueous solution was purged with dry argon (5.0 Messer) for 5 min to remove oxygen from the electrochemical cell. CV parameters were set as follows: initial potential *E*_o_ = −300 mV, final potential *E*_e_ = 1300 mV, and current sampling time *t*_s_ = 500 ms. DPV voltammograms were registered in 0.1 M acetate and phosphate buffer solutions with pH ranging from 3 to 7, in a potential range from *E*_o_ = −300 mV to *E*_e_ = 1800 mV, using a step potential of *E*_s_ = 5 mV, waiting time *t*_w_ = 10 ms, pulse amplitude d*E* = 40 mV, and *t*_s_ = 500 ms.

### 2.9 Antioxidant Activity Assays

Purified celosianin and its oxidized forms were subjected to antioxidant activity measurements using the ABTS (Trolox equivalent antioxidant capacity (TEAC)), ferric reducing antioxidant power (FRAP), and oxygen radical absorbance capacity (ORAC) assays. Stock solutions of each pigment (0.5 mg/mL) were prepared in deionized water and further diluted according to the assay requirements with concentrations ranging from 0 to 100 μg/mL. Trolox was used as a reference compound for calibration curves, and gallic acid was used as the reference antioxidant. All measurements were performed using a Microplate Reader Infinite 200 (Tecan Austria GmbH, Grödig/Salzburg, Austria). The results were expressed as the IC_50_ value in the ABTS assay (mg/L) and in mmol TE/g DW (mmol Trolox per g of dry weight of the sample) in all assays.

The ABTS assay was conducted following the method published by Re et al.^28^ Prior to the measurements, 40 μL of 1 mM ABTS^+•^ was added to all tested samples and Trolox, and the plates were incubated for 30 min in the dark. Spectrophotometric measurements were performed at 734 nm and at 25 °C.

The FRAP assay was conducted following the procedure reported by Benzie & Strain^29^ with slight modifications. To all tested samples and Trolox, 133 μL of FRAP reagent composed of 10 mM TPTZ, 20 mM ferric chloride (FeCl_3_), and 300 mM sodium acetate buffer at pH 3.6 (in a ratio of 1:10:1, *v:v:v*) was added. The mixtures were then incubated for 10 min, and the absorbance was measured at 593 nm.

The ORAC assay was conducted following the procedure described by Huang et al.^30^ with some modifications. Briefly, 25 μL of tested samples (pigments, gallic acid, and Trolox at different dilutions) were added to experimental wells. Then, 150 μL of sodium fluorescein working solution (10 nM) was added. The plate was at 37 °C for 30 min, and the reactions were initiated by adding 25 μL of 240 mM AAPH followed by 10 s of shaking. Fluorescence was kinetically monitored, with data collected every minute. Excitation was performed at 485 nm with a 20 nm bandpass, and emission was measured at 528 nm with a 20 nm bandpass. ORAC values were calculated using the area under curve (AUC) and the net area under curve (net AUC) of the standards and samples, following the method described by Cao & Prior,^31^ and expressed as mmol TE/g DW.

### 2.10 Evaluation of Cytotoxic and Cardioprotective Activities of Celosianin Derivatives

#### 2.10.1 Cell Culture

Rat cardiac myoblasts (H9c2) were obtained from the American Type Culture Collection (ATCC CRL-1446). The cells were cultured in Dulbecco’s modified Eagle’s medium (DMEM, PAN Biotech, Germany) with a reduced sodium bicarbonate content (1.5 g/L, PAN BIOTECH, Germany) supplemented with 10% fetal bovine serum (FBS, Biowest, France) and 1% penicillin/streptomycin (PAA, Austria) at 37 °C, 5% CO_2_, and a humidified atmosphere. All experiments described below were carried out in triplicate.

#### 2.10.2 Cell Viability Assessment Using the alamarBlue Assay

Cell viability was assessed using the alamarBlue assay (Sigma-Aldrich). H9c2 cells were seeded in 96-well plates at a density of 5 × 1 × 10^3^ cells/well for 24 h. The cells were then treated with varying concentrations of celosianin and its oxidized products 17-decarboxy-neocelosianin and 2,17-bidecarboxy-xanneocelosianin (0.1 to 1000 μg/mL). Untreated cells served as the control (0 μg/mL pigment concentration). After 24 h of treatment, the culture medium was removed and replaced with 100 μL of fresh DMEM containing 5% (*v/v*) alamarBlue reagent (Sigma-Aldrich). The plates were incubated for 3 h, and then 100 μL of the supernatants was transferred to a black 96-well plate. Fluorescence was measured at an excitation wavelength of 544 nm and emission wavelength of 590 nm using a microplate reader (FluoStar Omega, BMG Labtech, Germany). The percentage of resazurin reduction was calculated from the results using the following formula
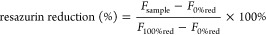
where:

*F*_sample_ is the fluorescence value of the sample.

*F*_0% red_ is the fluorescence of a medium containing unreduced alamarBlue reagent.

*F*_100% red_ is the fluorescence of a medium containing reduced alamarBlue reagent.

#### 2.10.3 Cell Morphology Determination Based on Fluorescence Live/Dead Imaging

To assess cell morphology, the media were removed from plates containing H9c2 cells, and 100 μL of calcein AM and propidium iodide solution (0.1% *v/v* each dissolved in phosphate-buffered saline (PBS), with all reagents from Sigma-Aldrich) was added. After a 10-min incubation in the dark, cells were imaged using a Zeiss Axiovert 40 fluorescence microscope (magnification 100×) with an HXP 120 C metal halide illuminator (Carl Zeiss, Germany).

#### 2.10.4 Caspase-3 Assay

To assess whether oxidized celosianins can prevent apoptosis in H9c2 cells, caspase-3 activity was measured using BioTracker NucView 530 Red Caspase-3 Dye (PBS) from Sigma-Aldrich. Cells were seeded in 96-well black plates with transparent flat bottoms at a density of 1 × 10^4^ cells/well. After 24 h, the cells were treated overnight with celosianin and its oxidation products (17-dNCel and 2,17-dXNCel) at the following concentrations: 0.1, 1.0, 10.0, 100.0, and 1000.0 μg/mL. Subsequently, the supernatants were replaced with 80 nM Paclitaxel (PAC) from Jiangsu Yew Pharmaceutical Co., Ltd., China, chosen as an inducer of apoptosis. The control consisted of cells grown in DMEM containing only PAC. Caspase-3 enzyme activity was measured according to the manufacturer’s guidelines for all samples and the control after 24 h of incubation. Fluorescence was measured after the addition of 5 μM reagent per well followed by a 30 min incubation at 37 °C using a BMG Labtech spectrofluorometer (FluoStar Omega; excitation: 523 nm, emission: 563 nm).

#### 2.10.5 GSH/GSSG Ratio Detection Assay

The effect of celosianin, 17-decarboxy-neocelosianin, and 2,17-bidecarboxy-xanneocelosianin on glutathione levels in H_2_O_2_-injured H9c2 cells was measured using the GSH/GSSG Ratio Detection Assay Kit (Fluorometric Green) (ab138881). Rat cardiomyoblasts were seeded at a density of 1 × 10^4^ cells/well in 96-well transparent plates. After 24 h, the cells were treated with the tested compounds in a concentration range of 0.1 to 1000 μg/mL and incubated overnight. Subsequently, DMEM media containing betalains were replaced with 500 μM hydrogen peroxide (H_2_O_2_) from Sigma-Aldrich, Germany, chosen as an inducer of oxidative stress. H9c2 cells in DMEM containing only 500 μM H_2_O_2_ served as controls. Glutathione solutions (0–10 μM) were used to generate the calibration curve. The intensity of the fluorescence signal, measured at an excitation of 490 nm and an emission of 520 nm using a BMG Labtech, FluoStar Omega spectrofluorometer, was directly proportional to the measured glutathione level.

### 2.11 Statistical Analysis

All experiments were carried out in triplicate, and the data are expressed as mean values ± standard deviation of three or more independent experiments. The statistical significance among the tested samples was determined in one-way analysis of variance (ANOVA) with Tukey’s post hoc test at a significant level of *p* < 0.05. The analyses were calculated using STATISTICA, version 10 (StatSoft, Inc. 2011), and OriginPro 2020.

## 3 Results and Discussion

### 3.1 Overview of Oxidation Mechanisms of Betanin- and Gomphrenin-Type Betacyanins

Preliminary studies on the enzymatic and nonenzymatic oxidation of betanin, betanidin, and neobetanin, as well as their decarboxylated derivatives and intermediate products, were performed by Wybraniec et al.^14,15,32^ Based on established pathways of DOPA and dopamine oxidation, betanidin, the aglycone of betacyanins, was found to likely undergo conversion into three tautomeric quinoid derivatives: *o*-quinone intermediate, quinone methide, and dopachrome derivative. This transformation leads to the formation of decarboxylated and dehydrogenated derivatives.^14,32^ The oxidation of betanin (betanidin 5-*O*-β-glucoside) produces a quinone methide intermediate that can rearrange to form 2,3-dehydro- (xan-) or 14,15-dehydro- (neo-) derivatives derivatives.^15^ Gomphrenin (betanidin 6-*O*-β-glucoside) generates only a dopachromic derivative as the quinonoid intermediate during oxidation. Furthermore, the oxidation of gomphrenin by the ABTS cation radical reveals that unlike betanin, the formation of its aglycone (betanidin) and its derivatives is also detected during oxidation. The results demonstrate that the glucosylation position of betanidin, specifically at 5-*O* and 6-*O*-, significantly influences its reactivity.^17^

Additionally, metal cations like copper, iron, aluminum, or tin can accelerate the degradation/oxidation of betacyanins, which is noteworthy when considering the potential release of metals from food packaging, such as cans.^33^ Decarboxylated and dehydrogenated products resulting from the oxidation of betanin-based pigments by the ABTS cation radical and Cu^2+^ complexation were isolated from reaction mixtures, purified, and structurally confirmed using NMR and HRMS. The results demonstrate that Cu^2+^-catalyzed oxidation of betanin yields neo-derivatives, while the oxidation of 17-decarboxy- and 2,17-bidecarboxy-betanin leads to the formation of xan-derivatives. Notably, it has been discovered that Cu^2+^-catalyzed oxidation of betanin can occur in the dihydropyridinic ring, potentially omitting the quinone methide stage in the dihydroindolic system.^16^ To expand on previous findings, this study investigated, for the first time, the oxidation of a distinct group of betacyanins that contain a glucuronosylglucosyl moiety.

### 3.2 Oxidation of Amaranthin-Type Betacyanins: LC-DAD-ESI-MS/MS and LC-Q-Orbitrap-MS Identification of Decarboxylated and Dehydrogenated Derivatives

#### 3.2.1 Identification of Amaranthin-Type Betacyanins Isolated from *A. hortensis'Rubra'* for the Oxidation Experiments

Amaranthin (betanidin 5-*O*-(2′-*O*-*β*-D-glucuronopyranosyl)-*β*-d-glucopyranoside) **2** is a common betacyanin with a characteristic glucuronosylglucosyl moiety attached to betanidin at position C-5. Furthermore, argentianin (betanidin 5-*O*-[(2′′-*O*-*E*-4-coumaroyl)-2′-*O*-*β*-d-glucuronopyranosyl]-*β*-d-glucopyranoside) **10** and celosianin (betanidin 5-*O*-[(2′′-*O*-*E*-feruloyl)-2′-*O*-*β*-d-glucuronopyranosyl]-*β*-d-glucopyranoside) **17** have previously been identified as amaranthin acylated with the *p*-coumaric and ferulic moieties, respectively.^2,3,34^ In this research, the initial identification of betacyanins and their oxidation products was performed using liquid chromatography coupled with low-resolution mass spectrometry (LC-DAD-ESI-MS/MS) based on the *m*/*z* signals of protonated molecular and fragmentation ions. The chromatographic, spectrophotometric, and mass spectrometric data of the substrates and the resulting oxidized products from ABTS oxidation are presented in Table 1. Additional confirmation of the molecular formulas and the fragmentation patterns of the oxidation products was obtained through high-resolution mass spectrometric measurements using an Orbitrap instrument (Table S1).

**Table 1:** Chromatographic, Spectrophotometric, and Mass Spectrometric Data of the Analyzed Products of Amaranthin, Argentianin, and Celosianin Oxidation by ABTS Radicals

no.	abbrev.	compound	*R*_t_ [min]		λ_max_ [nm]			*m*/*z* [M + H]^+^			*m*/*z* from MS/MS of [M + H]^+^	
Amaranthin Oxidation												
**1**	2,3-OH-2dAm	2,3-dihydroxy-2-decarboxy-xanamaranthin	4.0		527		715			539; 377; 359		
**1′**	2,3-OH-2dIAm	2,3-dihydroxy-2-decarboxy- -xanisoamaranthin	4.5		527		715			539; 377; 359		
**2**	Am	amaranthin	4.7		534		727			551; 389; 343; 297; 255		
**2′**	IAm	isoamaranthin	5.2		534		727			551; 389; 343; 297; 255		
**3**	2-dXAm	2-decarboxy-xanamaranthin	7.4		445		681			505; 459; 343		
**4**	2,17-dXAm	2,17-bidecarboxy-xanamaranthin	7.8		427		637			461; 417		
**5**	17-dNAm	17-decarboxy-neoamaranthin	7.9		480		681			505; 459; 343		
**6**	2,17-dXNAm	2,17-bidecarboxy-xanneoamaranthin	9.6		412		635			459		
**7**	2,17-dNAm	2,17-bidecarboxy-neoamaranthin	10.2		452		637			461; 417		
**8**	2-dXNAm	2-decarboxy-xanneoamaranthin	12.3		422		679			503; 459; 341; 297; 253		
Argentianin Oxidation												
9	2,3-OH-2dArg	2,3-dihydroxy-2-decarboxy-xanargentianin		7.5		531			861			539; 377; 359
9′	2,3-OH-2dIArg	2,3-dihydroxy-2-decarboxy- -xanisoargentianin		7.5		531			861			539; 377; 359
10	Arg	argentianin		8.3		540			873			551; 389; 343; 297; 255
10′	IArg	isoargentianin		8.7		540			873			551; 389; 343; 297; 255
11	17-dNArg	17-decarboxy-neoargentianin		11.2		450			827			681; 505; 343;297
12	2,17-dXArg	2,17-bidecarboxy-xanargentianin		11.8		465			783			637; 461; 417; 299; 253
13	2,17-dXNArg	2,17-bidecarboxy-xanneoargentianin		13.3		426			781			459; 297; 251
14	2,17-dNArg	2,17-bidecarboxy-neoargentianin		13.4		426			783			637; 461; 417; 299; 253
15	2-dXNArg	2-decarboxy-xanneoargentianin		15.9		435			825			679; 503; 459; 341; 295; 251
Celosianin Oxidation												
16	2,3-OH-2dCel	2,3-dihydroxy-2-decarboxy-xancelosianin		8.0	532				891			873; 829; 539; 521; 377; 359
16′	2,3-OH-2dICel	2,3-dihydroxy-2-decarboxy- -xanisocelosianin		8.1	532				891			873; 829; 539; 521; 377; 359
17	Cel	celosianin		8.8	541				903			551; 389; 343; 297; 253
17′	ICel	isocelosianin		9.0	541				903			551; 389; 343; 297; 253
18	17-dNCel	17-decarboxy-neocelosianin		11.4	452				857			681; 505; 459; 343; 297; 253
19	2,17-dXCel	2,17-bidecarboxy-xancelosianin		11.6	460				813			461; 417; 299; 253
20	2,17-dXNCel	2,17-bidecarboxy-xanneocelosianin		13.1	415				811			459; 297; 251
21	2,17-dNCel	2,17-bidecarboxy-neocelosianin		13.5	466				813			461; 417; 299; 253
22	2-dXNCel	2-decarboxy-xanneocelosianin		15.6	432				855			503; 459; 341; 295

Amaranthin **2**, argentianin **10**, and celosianin **17** yield ions at *m*/*z* 727.1830, 873.2198, and 903.2306, respectively, with confirmed molecular formulas of C_30_H_35_N_2_O_19_ (calculated *m*/*z*: 727.1829), C_39_H_41_N_2_O_21_ (calculated *m*/*z*: 873.2196), and C_40_H_43_N_2_O_22_ (calculated *m*/*z*: 903.2302). These compounds share a common pattern in HCD experiments in positive ionization mode. The detachment of a glucuronosyl moiety produces fragment ions [M + H]^+^ at *m*/*z* 551, corresponding to betanin. Furthermore, the fragmentation of betanin generates a daughter ion at *m*/*z* 389, resulting from a neutral loss of a glucosyl residue (−162 Da). Additional characteristic daughter ions detected at *m*/*z* 343 and 297 arise from a monodecarboxylation and dehydrogenation (343 = 389–46) as well as double decarboxylation and double dehydrogenation (297 = 389–92) of betanidin ions (*m*/*z* 389). In the case of amaranthin and argentianin, triple decarboxylation and mono dehydrogenation (255 = 389–134) occur in the betanidin ion (*m*/*z* 389), while the fragmentation of celosianin results in the formation of a triple decarboxylated and double dehydrogenated derivative (253 = 389–136). No detachment of *p*-coumaroyl or feruloyl moieties was detected within the argentianin and celosianin structures (Tables 1 and S1).

#### 3.2.2 Identification of the First Oxidation Products of Amaranthin-Type Betacyanins

**Figure 1:**
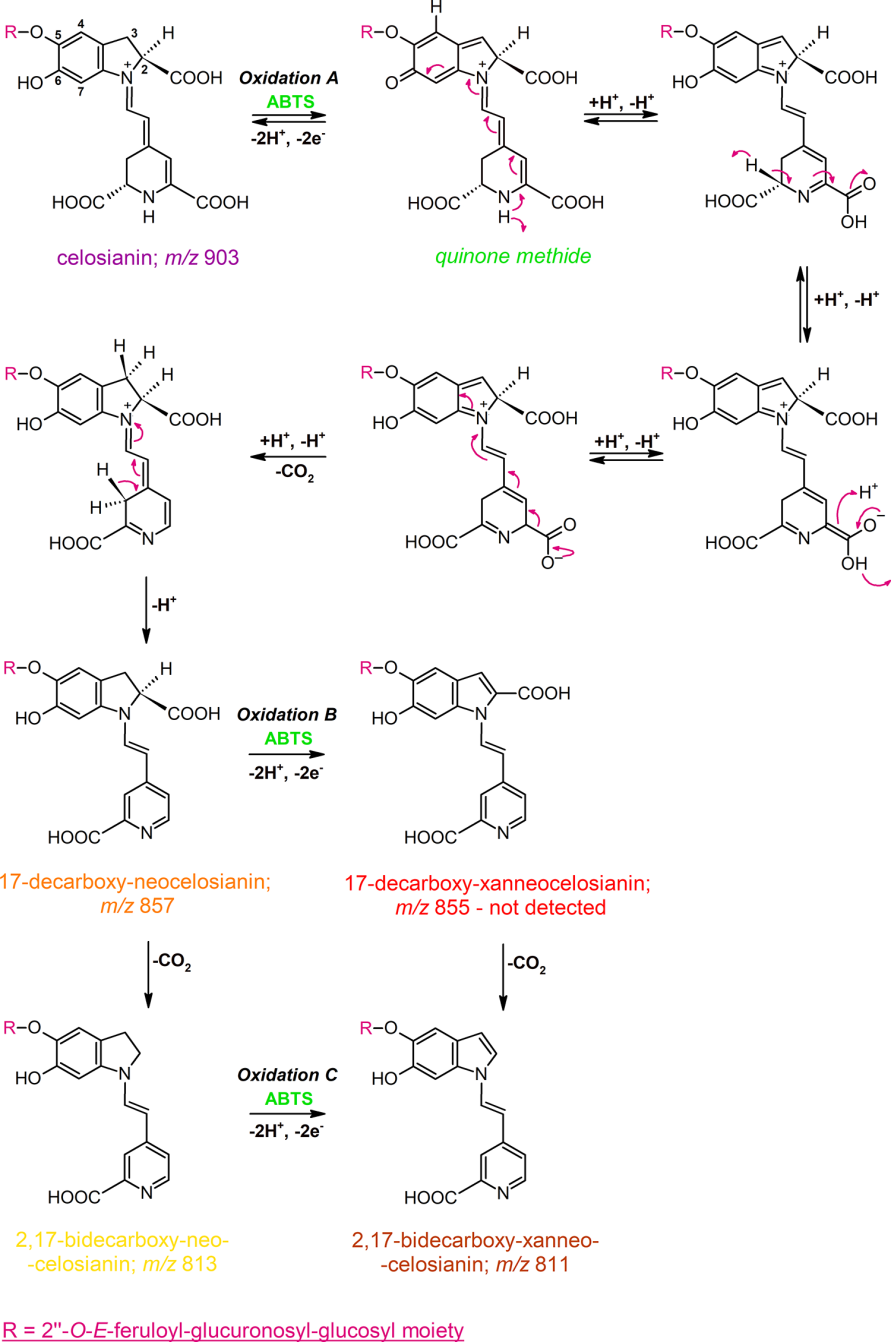
Proposed mechanism for the oxidation of celosianin **17** by ABTS cation radical, resulting in the formation of neo-derivatives as the main oxidation products: 17-decarboxy-neocelosianin **18**, 2,17-bidecarboxy-neocelosianin **21**, and 2,17-bidecarboxy-xanneocelosianin **20**, with a quinone methide intermediate. The same mechanism is proposed for the oxidation of amaranthin and argentianin.

**Figure 2:**
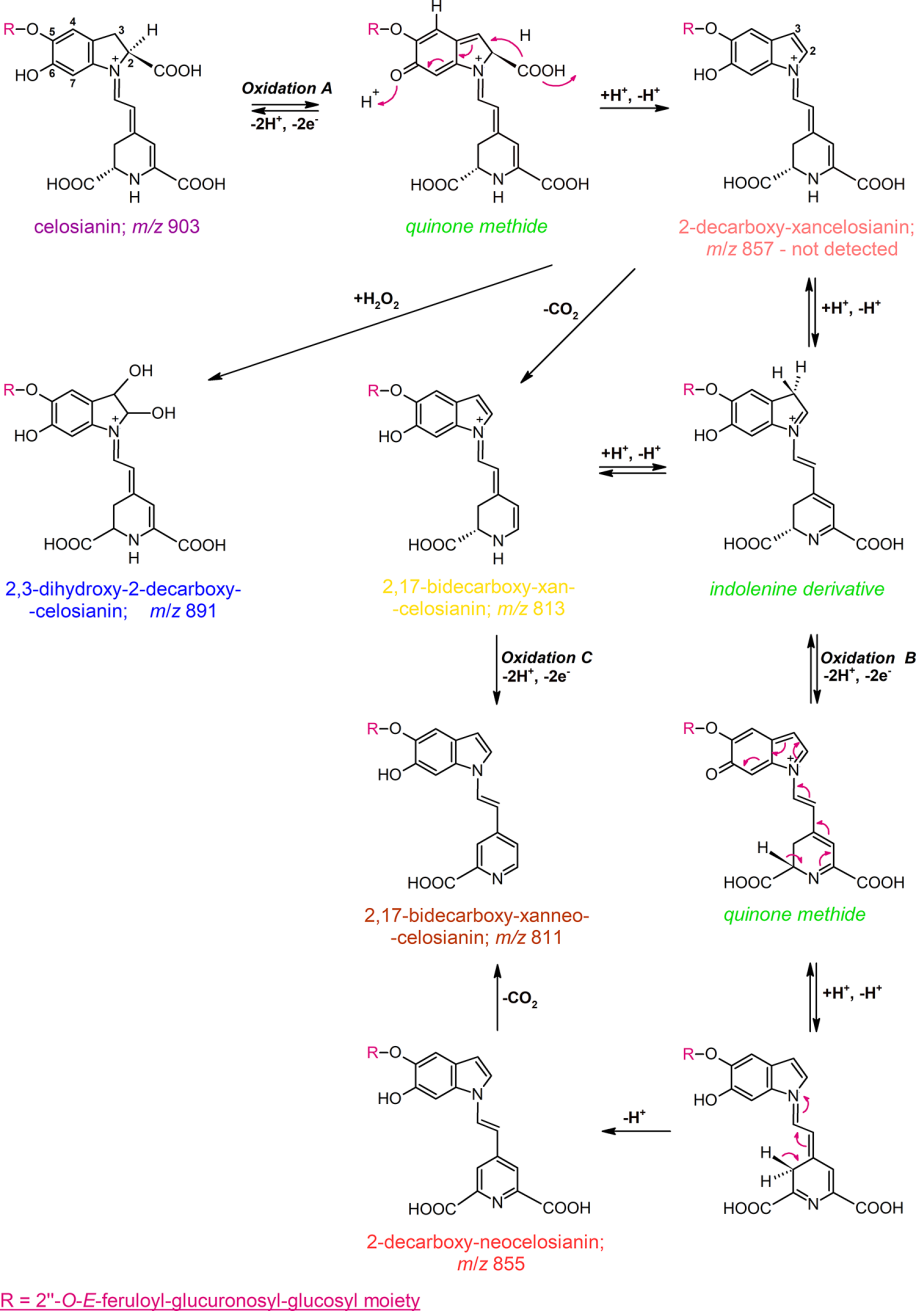
General scheme of possible transformations of celosianin **17** during ABTS cation radical oxidation, leading to the generation of xan-derivatives: 2-decarboxy-xanneocelosianin **22**, 2,17-bidecarboxy-xancelosianin **19**, and 2,17-bidecarboxy-xanneocelosianin **20**. The same mechanism is proposed for the oxidation of amaranthin and argentianin.

**Figure 3:**
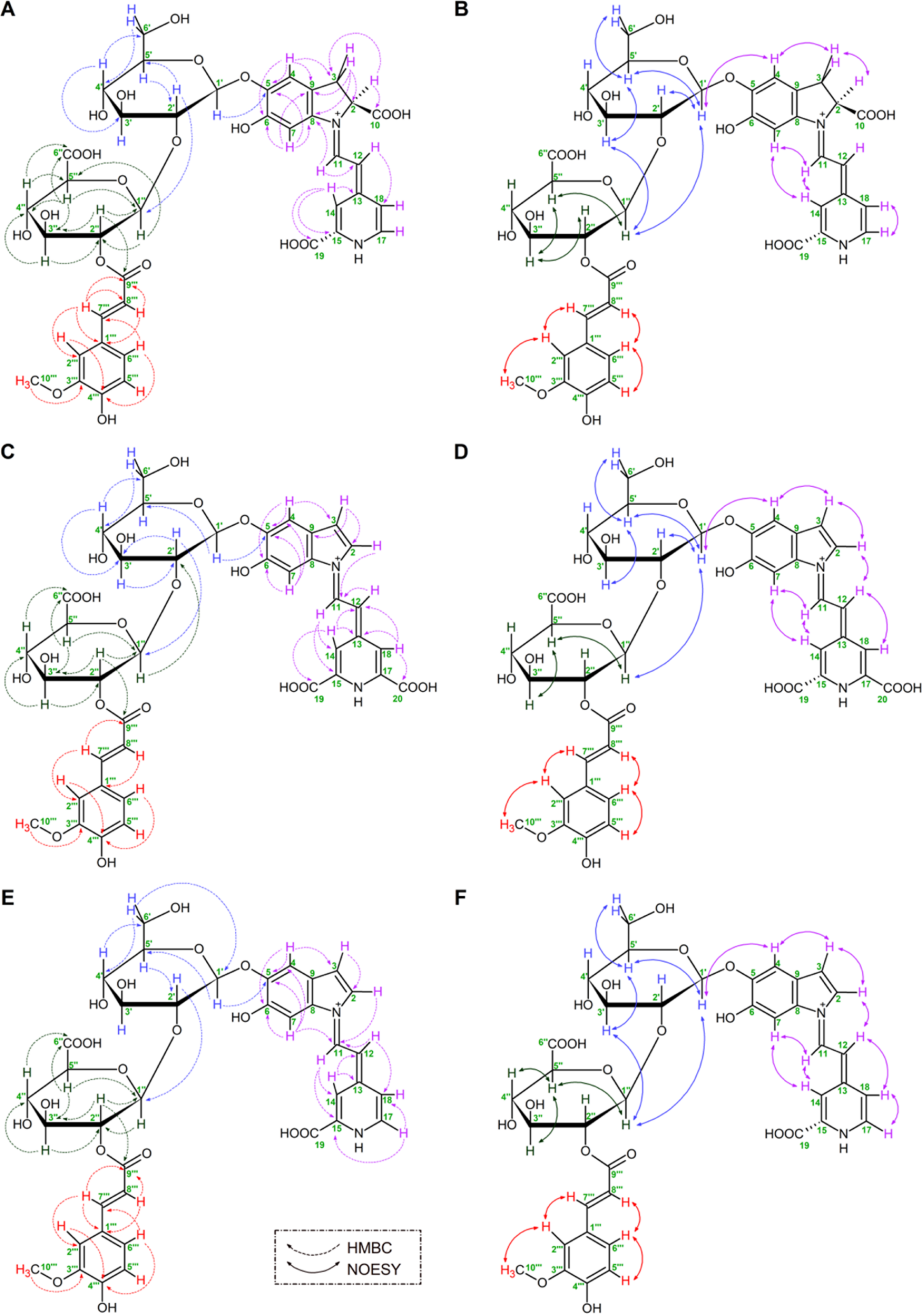
Significant HMBC and NOESY NMR correlations in the structures of 17-decarboxy-neocelosianin **18** (A, B), 2-decarboxy-xanneocelosianin **22** (C, D), and 2,17-decarboxy-xanneocelosianin **20** (E, F). ^1^H NMR and ^13^C spectra can be consulted in the Supporting Information.

Oxidation experiments were conducted on amaranthin **2**, argentianin **10**, and celosianin **17** using the ABTS cation radical at pH ranging from 3 to 7.4 for 80 min. The highest oxidation activity was observed in an acidic environment at pH 3 and 4, consistent with the oxidation of betanin and gomphrenin but contrasting with betanidin. Figure S1 presents the chromatographic traces of the main oxidation products obtained after 80 min of celosianin oxidation, which were subsequently subjected to *in vitro* tests (Sections 3.6 and 3.7). Based on LC-MS/MS detection, the main products of amaranthin **2**, argentianin **10**, and celosianin **17** oxidation were characterized by protonated molecular ions [M + H]^+^ at *m*/*z* 681, 827, and 857, respectively. This suggests the initial formation of monodecarboxylated and monodehydrogenated derivatives (Table 1). The fragmentation patterns for the oxidation products were similar to those of their respective precursors. High-resolution mass spectrometric (HRMS) analyses determined the molecular formulas of C_29_H_33_N_2_O_17_, C_38_H_39_N_2_O_19_, and C_39_H_41_N_2_O_20_ for the protonated molecular ions at *m*/*z* 681.1772, 827.2122, and 857.2237, respectively, providing further support for the presence of monodecarboxylated and monodehydrogenated products of amaranthin, argentianin, and celosianin, respectively. The fragmentation of the parent ions of *m*/*z* 681, 827, and 857 resulted in the detection of *m*/*z* signals at 505, indicating detachment of the glucuronosyl moiety, as well as the *m*/*z* signal at 343 corresponding to the neutral loss of the glucosyl moiety from the oxidation products. Interestingly, fragmentation of the *E-p*-coumaroyl and *E*-feruloyl moieties was detected within monodecarboxylated and monodehydrogenated ions of argentianin and celosianin. The LC-MS/MS and LC-Q-Orbitrap-MS analyses of the oxidation products of amaranthin-type betacyanins, along with NMR confirmation of celosianin derivative structures (Tables 1, 2, and S1 and Figures 3 and S2–S7), revealed the presence of 17-decarboxy-neobetacyanins **5**, **11**, and **18** as the main oxidation products of amaranthin **2**, argentianin **10**, and celosianin **17**, respectively. This suggests an influence of the glucuronosylglucosyl moiety linked with the acyl group in the oxidation of the dihydropyridinic system (Figures 1–3). For amaranthin **2**, however, the two oxidation pathways were observed, resulting in the formation of not only 17-decarboxy-neoamaranthin **5** (*m*/*z* 681.1772) but also 2-decarboxy-xanamaranthin **3** (*m*/*z* 681.1771). Unlike argentianin and celosianin, the main products of amaranthin oxidation, 2-decarboxy-xanamaranthin **3** is stable enough to be detected by LC-MS/MS (Tables 1 and S1). The proposed chemical structure of 2-decarboxy-xanamaranthin **3** is further supported by its characteristic maximum absorption wavelength of 445 nm, which is similar to the value previously obtained for 2-decarboxy-xanbetanin (λ_max_ = 446 nm).^15^ On the other hand, 17-decarboxy-neoamaranthin **5** is characterized by λ_max_ = 480 nm, and its corresponding analogous product in betanin oxidation experiments has never been detected.^14−16^ The presence of the glucuronosyl moiety attached to the sugar unit in the tested derivative may presumably exhibit a stabilizing effect on 17-decarboxy-neoamaranthin **5**.^14,15^

**Table 2:** NMR Data Obtained for 17-Decarboxy-neocelosianin **18**, 2,17-Decarboxy-xanneocelosianin **20**, and 2-Decarboxy-xanneocelosianin **22** Structures

	17-dNCel **18** (D_2_O)		2-dXNCel **22** (DMSO/d-TFA)		2,17-dXNCel **20** (DMSO/d-TFA)	
no	^1^H NMR^a^	^13^C NMR^b^^c^	^1^H NMR^a^	^13^C NMR^b^^,^^c^	^1^H NMR^a^	^13^C NMR^b^^,^^c^
2	4.33, dd, 4.0; 10.8	64.4	7.76, d, 3.4	123.0	7.78, d, 3.6	123.0
3a/b	3.03, dd, 16.4; 3.8	34.1	6.63, d, 3.2	107.0	6.75, d, 3.3	109.8
	3.51, dd, 10.3; 16.2					
4	6.94, (overlap)	111.9	7.37, s	109.1	7.39, s	109.3
5		141.6		142.6		143.1
6		146.5		145.5		145.8
7	6.42, bs	98.9	7.74, s	97.6	7.80, s	98.2
8		138.6		133.0		142.2
9		122.1		121.1		121.8
10		178.4				
11	7.51, d, 13.3	138.4	8.55, d, 14.7	130.4	8.83, d, 14.3	135.5
12	5.26, d, 13.4	98.5	7.04, d, 14.3	108.5	7.16, d, 14.2	106.5
13		161.4		159.4		161.3
14	7.86	133.0	8.46, s	123.4	8.64, s	122.2
15		150.1		148.3		155.8
17	7.93, d, 8,7 (overlap)	138.8		148.3	8.69, d, 6.2	142.2
18	6.96 (overlap)	123.0	8.46, s	123.4	8.24, d, 5.7	123.5
19		172.0		165.8		169.6
20				165.8		
1′	5.51, d, 8.1	96.3	4.70, d, 7.7	102.5	4.72, d, 8.0	102.5
2′	4.00, m	73.2	3.63, m	79.3	3.64, m	79.2
3′	3.59 (overlap)	76.4	3.33 (overlap)	77.0	3.34 (overlap)	76.6
4′	3.57, (overlap)	70.2	3.19, m	70.2	3.18, m	69.8
5′	3.87, (overlap)	80.6	3.31 (overlap)	77.0	3.32 (overlap)	75.9
6′a/b	3.73 (overlap)	61.2	3.49 (overlap)	60.8	3.48 (overlap)	60.7
	3.88 (overlap)		3.72, m, 10.6		3.72, m, 10.6	
1″	5.30, d, 7.9	96.7	5.05, d, 8.1	101.2	5.06, d, 8.1	101.1
2″	4.91, m	74.2	4.73, t, 8.8	73.6	4.73, (overlap)	73.5
3″	3,78, (overlap)	75.1	3.49 (overlap)	73.9	3.49 (overlap)	73.6
4″	3.68, (overlap)	72.2	3.51 (overlap)	71.8	3.50 (overlap)	71.4
5″	3.94, m	76.4	3.82 (overlap)	75.7	3.83 (overlap)	75.3
6″		175.8		175.7		175.0
1‴		127.6		125.8		125.8
2‴	6.95 (overlap)	111.0	7.27, bs	111.3	7.27, d, 1.7	111.3
3‴		148.5		148.7		147.9
4‴		148.9		149.2		149.2
5‴	6.74, d, 8.1	116.2	6.79, d, 8.2	115.6	6.79, d, 8.2	115.5
6‴	6.88, d, 7.2	124.1	7.10, d, 8.1	123.0	7.10, dd, 8.2; 1.6	122.9
7‴	7.22, d, 15.9	146.3	7.53, d, 15.7	144.8	7.52, d, 15.7	144.8
8‴	5.96, d, 15.9	113.6	6.41, d, 15.7	115.1	6.44, d, 15.9	115.0
9‴		169.0		165.9		165.8
10‴	3.80, s (overlap)	56.5	3.81, s (overlap)	55.8	3.81, s (overlap)	55.7

a ^1^H NMR δ [ppm], mult, *J* [Hz].

b ^13^C NMR δ [ppm].

c ^13^C chemical shifts were derived from *g*HSQC and *g*HMBC; *b* – broad signal, *s* – singlet, *d* – doublet, *t* – triplet, *dd* – double doublets, *m* – multiplet.

#### 3.2.3 Nonoxidative Decarboxylation of the First Oxidation Products of Amaranthin-Type Betacyanins

Simultaneously, 17-decarboxy-neo-derivatives **5**, **11**, and **18** of amaranthin **2**, argentianin **10**, and celosianin **17**, respectively, may undergo nonoxidative decarboxylation, resulting in the formation of 2,17-bidecarboxy-neo-derivatives **7**, **14**, and **21**, respectively. Additionally, the reaction mixtures contained 2,17-bidecarboxy-xan-derivatives **4**, **12**, and **19** (Table 1). These compounds exhibited parent ions [M + H]^+^ at *m*/*z* 637, 783, and 813, respectively. The fragmentation ions at *m*/*z* 461 and 299 confirmed the presence of bidecarboxylated and monodehydrogenated fragments, resulting from the neutral loss of the glucuronosyl and the glucuronosylglucosyl moieties, respectively. Further fragmentation of xan-derivatives **4**, **12**, and **19**, and neo-derivatives **7**, **14**, and **21** ions was also combined with decarboxylation and dehydrogenation steps (Table S1).

#### 3.2.4 Identification of the Final Oxidation Products of Amaranthin-Type Betacyanins

Subsequent oxidation of the 2,17-bidecarboxy-neo-derivatives **7**, **14**, and **21** led to the formation of the final oxidation products, 2,17-bidecarboxy-xanneo-derivatives **6**, **13**, and **20**, respectively. These compounds retained the chromophoric system and exhibited protonated molecular ions at *m*/*z* 635, 781, and 811, respectively (Table 1). Fragmentation analysis revealed a pattern similar to that of the previously mentioned ions, resulting in the loss of the glucuronosyl (−176 Da) and glucuronosylglucosyl (−338 Da) moieties. Further fragmentation steps for 2,17-bidecarboxy-xanneoargentianin **13** and 2,17-bidecarboxy-xanneocelosianin **20** involved decarboxylation, yielding final diagnostic ions at *m*/*z* 253.

#### 3.2.5 Identification of Monodecarboxylated Xanneo-Derivatives of Amaranthin-Type Betacyanins

Monodecarboxylated and bidehydrogenated (xanneo-) derivatives, which displayed the longest retention times, were identified as the most hydrophobic oxidation products, consistent with the previous studies.^14,15,32^ The compounds identified as 2-decarboxy-xanneo-derivatives **8, 15, and 22** exhibited parent ions [M + H]^+^ at *m*/*z* 679.1619, 825.1988, and 855.2088, respectively (Table S1) and produced fragmentation ions at *m*/*z* 503 and 341 resulting from the neutral loss of the glucuronosyl moiety (−176 Da) and the glucuronosylglucosyl moiety (−338 Da), respectively. Additionally, detachment of the *p*-coumaroyl moiety was observed for 2-decarboxy-xanneoargentianin **15**, giving the fragmentation ion at *m*/*z* 679.2051. These findings were further supported by the NMR structural elucidation of 2-decarboxy-xanneocelosianin (Table 2 and Figures 3 and S2–S7). Additionally, the presence of 17-decarboxy-xanneo-derivatives was not detected in the reaction mixtures. Figures 1 and 2 present a proposed scheme illustrating the possible oxidation pathways for acylated betacyanins in the presence of the ABTS cation radical.

#### 3.2.6 Tentative Identification of Dihydroxylated Derivatives of Oxidized Products

Further inspection of the chromatograms revealed the presence of more polar pigments tentatively identified as dihydroxylated derivatives of monodecarboxylated and monodehydrogenated amaranthin-type betacyanins. These compounds exhibited protonated molecular ions [M + H]^+^ at *m*/*z* 715.1831, 861.2198, and *m*/*z* 891.2305 for dihydroxylated decarboxy-dehydro-amaranthin **1** (C_29_H_35_N_2_O_19_), argentianin **9** (C_38_H_41_N_2_O_21_), and celosianin **16** (C_39_H_43_N_2_O_22_), respectively. LC-Q-Orbitrap-MS analysis confirmed these findings, displaying fragmentation ions at *m*/*z* 539 (loss of feruloyl and glucuronosyl moieties), *m*/*z* 377 (loss of feruloyl and glucuronosylglucosyl moieties), and *m*/*z* 359 (loss of H_2_O from ion at *m*/*z* 377). Fragmentation of dihydroxylated decarboxy-dehydro-celosianin (*m*/*z* 891) yielded ions at *m*/*z* 873, 829, and 521 derived from neutral loss of H_2_O (−18 Da), loss of H_2_O combined with decarboxylation (−62 Da), and subsequent detachment of feruloyl and glucuronosyl moieties (−352 Da). The detected novel pigments likely originate from the generation of 2-decarboxy-2,3-dehydro-derivatives during oxidation, followed by their reaction with hydroxyl radicals formed during the action of ABTS^+•^. Although the exact position of the attachment of the two hydroxyl groups (+34 Da) is yet to be determined, the previous report suggests that hydroxylation likely occurs at carbon position C-2,3.^15^

#### 3.2.7 Mechanism of ABTS Cation Radical-Mediated Oxidation of Amaranthin and Acylated Amaranthins Resulting in Neo-Derivative Formation

The identification of oxidation products in amaranthin-type betacyanins provides valuable insights into the underlying chemical reactions. Based on oxidation experiments with betanin, where the presence of the glucosyl moiety blocks the phenolic group at carbon atom C-5, it was expected that decarboxylation in acylated betacyanins occurs at carbon atom C-2. This specific decarboxylation process could be associated with the conversion of a quinone methide intermediate to an indolic derivative, ultimately resulting in the formation of xan derivatives.

Interestingly, celosianin oxidation proceeds via two pathways (Figures 1 and 2), which differ in some aspects from previous reports.^14−16^ Unlike betanidin and betanin, celosianin undergoes a single decarboxylation process during oxidation, but it occurs at carbon atom C-17 instead of the expected carbon atom C-2. This results in the formation of 17-decarboxy-neocelosianin **18** (*m*/*z* 857) as a main oxidation product. The formation of 17-dNCel **18**, the most polar oxidation product, involves the generation of a celosianin quinone methide, followed by rearrangement of the conjugated system in the aglycone (dihydropyridinic part of the core), decarboxylation at carbon C-17, and aromatization of the dihydropyridinic system (Figure 1, Oxidation A).

Further decarboxylation of 17-decarboxy-neocelosianin **18** occurs at the carbon atom C-2, leading to the formation of 2,17-bidecarboxy-neocelosianin **21** (*m*/*z* 813). Subsequently, an additional oxidation step results in the generation of 2,17-bidecarboxy-xanneocelosianin **20** (*m*/*z* 811) (Figure 1, Oxidation C). This process is hypothesized to involve the formation of a quinone methide from 2,17-dNCel **21**, which contains the 6-*O*-phenolic group, followed by rearrangement to the xanneo-derivative **20**. However, the alternative pathway, which involves the oxidation of 17-dNCel **18** to 17-decarboxy-xanneocelosianin (*m*/*z* 855), followed by decarboxylation at C-2 and the generation of the final product 2,17-dXNCel **20**, is unlikely as 17-dXNCel was not detected in the reaction mixture (Figure 1, Oxidation B). The same pathways may be involved in the oxidation of amaranthin and argentianin.

#### 3.2.8 ABTS Cation Radical-Mediated Oxidation Mechanism for Xan-Derivative Formation in Amaranthin and Acylated Amaranthins

Unexpectedly, the anticipated oxidation product of celosianin, 2-decarboxy-xancelosianin (*m*/*z* 857), was not present in the reaction mixtures, contrasting the results obtained for betanin, its decarboxylated derivatives,^15^ and amaranthin in the current study (Figure 2, Oxidation A). The possible instability of 2-dXCel suggests its conversion to other oxidation products. This is supported by the tentative identification of its decarboxylated derivative, 2,17-bidecarboxy-xancelosianin **19** (*m*/*z* 813), as well as the doubly oxidized pigment 2,17-bidecarboxy-xanneocelosianin **20** (*m*/*z* 811) (Figure 2, Oxidation C). Additionally, the structures of these compounds were confirmed through NMR analyses (Table 2 and Figures S2–S7).

The 2,3-dehydrogenation pathway (Figure 2, Oxidation B) is further supported by the rearrangement of the unstable 2-decarboxy-xancelosianin (*m*/*z* 857) to a neo-derivative via quinone methide. The presence of a monodecarboxylated and doubly dehydrogenated celosianin derivative (*m*/*z* 855) was confirmed through mass spectrometry, and its structure as 2-decarboxy-xanneocelosianin **22** was determined through NMR analysis (Table 2 and Figures S2–S7). The other possible transformation of 2-dXCel is the formation of its 2,3-dihydroxylated derivative **9** (*m*/*z* 891), which corresponds to the chromatographic peak at 8.5 min (Table 1). It was previously concluded that the generation of dihydroxylated derivatives of oxidized betalains could result from hydroxylation by H_2_O_2_.^15^

Analogous oxidation pathways are also proposed for argentianin and amaranthin. However, the low quantities of the key compounds prevented further detailed NMR analysis of the corresponding products.

### 3.3 NMR Structural Elucidation of Decarboxylated and Dehydrogenated Celosianins

The structures of the three oxidation products of celosianin, namely, 17-decarboxy-neocelosianin **18**, 2,17-bidecarboxy-xanneocelosianin **20**, and 2-decarboxy-xanneocelosianin **22**, were determined using one- and two-dimensional NMR techniques. Deuterium oxide (D_2_O) was selected as the solvent for long-term NMR measurements of 17-decarboxy-neocelosianin **18**, as it showed no destructive effects.^35^ The structural analyses of the fully oxidized derivatives **20** and **22**, which exhibited greater stability compared to the non-oxidized precursor celosianin **17**, were performed in the DMSO/*d*-TFA solvent.^16^ This enabled the detection of distinct signals for the zwitterionic structures stabilized by *d*-TFA (Table 2 and Figures S2–S7). The appearance of NMR signals characteristic of the betanidin and glucosyl group confirmed the presence of celosianin-based cores with modified chromophoric systems^2^ within **18**, **20**, and **22**. This is consistent with previous data obtained for oxidized betanins.^16^ The presence of characteristic vinyl protons (H-11 and H-12) in the conjugated betacyanin system at low- and high-field positions, respectively, was observed in all three compounds, **18**, **20**, and **22**.

In the COSY and TOCSY spectra, individual coupled ^1^H-spin systems of H-2 and H-3a/b in **18**, as well as H-2 and H-3 in compounds **20** and **22**, were readily detected. This confirmed the presence of dehydrogenation at positions C-2 and C-3, as well as decarboxylation at carbon C-2 only, in compounds **20** and **22**. The absence of the coupled ^1^H-spin system of H-14a/b and H-15, previously observed in celosianin,^2^ confirmed the additional dehydrogenation at positions C-14 and C-15 in all of the oxidized pigments. Instead, the presence of a single proton H-14 was observed in the spectra, along with the detection of the aromatic proton H-18 in **18**, **20**, and **22**, and its coupled proton H-17 in compounds **18** and **22**. The presence of the latter proton indicated decarboxylation at position C-17 in **18** and **22**. These signals provided direct evidence of decarboxylation at positions C-2 and/or C-17, as well as dehydrogenation at positions C-2,3 and/or C-14,15, in the tested products **18**, **20**, and **22**. The presence of dihydroindolic systems (in compounds **20** and **22**) and an indolic system (in compound **18**) was confirmed by HSQC correlations of the protons in C-2, C-3, C-4, and C-7 with their respective carbons. In the pyridinic system, correlations of H-14 and H-18 in compound **20**, as well as H-14, H-17, and H-18 in compounds **18** and **22**, with their respective carbons, were observed in the HSQC spectra.

The presence of dihydroindolic systems (in compounds **20** and **22**) and an indolic system (in compound **18**) was further confirmed by using the HMBC technique. Correlations were observed between H-2 and C-3,11 for all of the analyzed pigments. Compound **18** exhibited additional cross-peaks: H-3ab to C-2,4,5,8,9,10,11; H-4 to C-3,5,6,8,9; and H-7 to C-5,6,8,9. For compounds **20** and **22**, cross-peaks were observed between H-3 and C-2,9,11; H-4 and C-3,5,6,7,8 as well as H-7 and C-4,5,6,9 (Table 2 and Figure 3).

Furthermore, the presence of the pyridinic system was indicated by the following HMBC correlations: in compound **18**, H-12 to C-18; H-14 to C-13,15,19; H-15 to C-13,15,19,14; H-17 to C-13; and H-18 to C-13; in compound **20**, H-11 to C-14,15,17,18; H-12 to C-14,18; H-14 to C-12,13,18,19; and H-18 to C-12,13,14,20; and in compound **22**, H-11 to C-14,15; H-12 to C-14,18; H-14 to C-12,13,18; H-17 to C-15,18; and H-18 to C-14 (Table 2 and Figure 3).

In the most abundant stereoisomers of compounds **18**, **20**, and **22**, the (*E*)-configuration for C(12)=C(13) and s-*trans* conformation for the dienyl moiety N(1)=C(11)–C(12)=C(13)^36^ were determined. This was supported by additional correlations among H-7, H-11, and H-14, as well as cross-peaks of H-2 and H-12 observed in the NOESY spectra (Figure 3). These findings are consistent with those previously observed in celosianin.^2^ Furthermore, NOESY correlations among proton systems H-2, H-3 (or H-3a/b in compound **18**), and H-4 were also observed (Figure 3).

The presence of the acylated glycosidic system characteristic of celosianin^2^ was initially indicated in compounds **18**, **20**, and **22** by the detection of the glucosyl and glucuronosyl moieties, with the two anomeric protons H-1′ and H-1″ observed in the HSQC spectrum. In the HMBC spectra, a correlation between the glucuronosyl proton H-2″ and the carbonyl carbon C-9‴ indicated that the trans-feruloyl moiety was attached to carbon C-2″ of the glucuronosyl moiety.

Furthermore, HMBC, TOCSY, and COSY correlations provided additional support for the presence of the two sugar rings (Table 2 and Figure 3). The correlation between proton H-2′ and carbon C-1″ indicated the linkage position of the second sugar group (glucuronosyl) as determined by HMBC. The β-linkages between the aglycone and glucopyranosyl,^35^ as well as between the glucopyranosyl and glucuronosyl moieties, were established based on the three-bond vicinal proton coupling constants (^3^*J*_1′-2′_ as well as ^3^*J*_1″-2″_ ∼8 Hz, respectively).

The glucosylation position of the aglyconic phenolic group (C-5) was established based on the HMBC correlation of H-1′ with C-5 and the NOESY correlation between H-1′ and H-4 (Figure 3) as described previously.^35^ The presence of the glucuronosyl ring within analyzed structures was confirmed by the HMBC detection of carbon C-6″ within the carboxyl moiety, which correlated with the protons H-5″ and H-4″ (Table 2 and Figure 3). Additional NOESY correlations between selected protons of the interlinked glucosyl and glucuronosyl moieties were also observed (Figure 3), similar to those previously established in the celosianin structure.^2^

The typical signals of the *E*-feruloyl moiety were observed in the ^1^H NMR spectra, including the olefinic protons (*J* ∼ 15.8 Hz, H-7‴ and H-8‴), aromatic protons (H-2‴, H-5‴, and H-6‴), and the signal at δ ∼ 3.8 ppm (H-10‴) from the methoxy group. This observation was supported by the ^13^C NMR spectra, which exhibited two olefinic carbon signals for C-7‴ and C-8‴, as well as an ester carbonyl carbon (C-9‴) along with the corresponding HMBC correlations (Figure 3). Additional NOESY cross-peaks for the protons of the *E*-feruloyl moiety, including H-2‴, H-5‴, H-6‴, H-7‴, H-8‴, and H-10‴, were also observed (Figure 3).

### 3.4 Electrochemical Studies of Amaranthin-Type Pigments

Electrochemical measurements are valuable for studying the physicochemical properties and metabolic pathways of antioxidants. Cyclic voltammetry (CV), differential pulse voltammetry (DPV), and square wave voltammetry (SWV) are commonly employed techniques to assess the electron-donating ability of compounds at the anodic peak potential. These techniques are also used to evaluate the scavenging activity and total antioxidant capacity of natural compounds, particularly polyphenols, in various food products.^37^

Understanding the precise mechanism of betacyanin oxidation is of great importance due to the high activity of these compounds.^15^ Betalain pigments, which possess the phenolic group in the dihydroindolic ring, can undergo electron transfer reactions and are amenable to investigation through electrochemical techniques such as DPV and CV. However, the electrochemical behavior of betacyanins^32^ and betaxanthins^38^ has been relatively underexplored, with only a limited number of studies conducted on this topic. We conducted additional experiments on a broader range of glucuronosylglucosylated betanidin derivatives to enhance the understanding of betalain electrochemistry.^32^

#### 3.4.1 Cyclic Voltammetry Measurements

**Figure 4:**
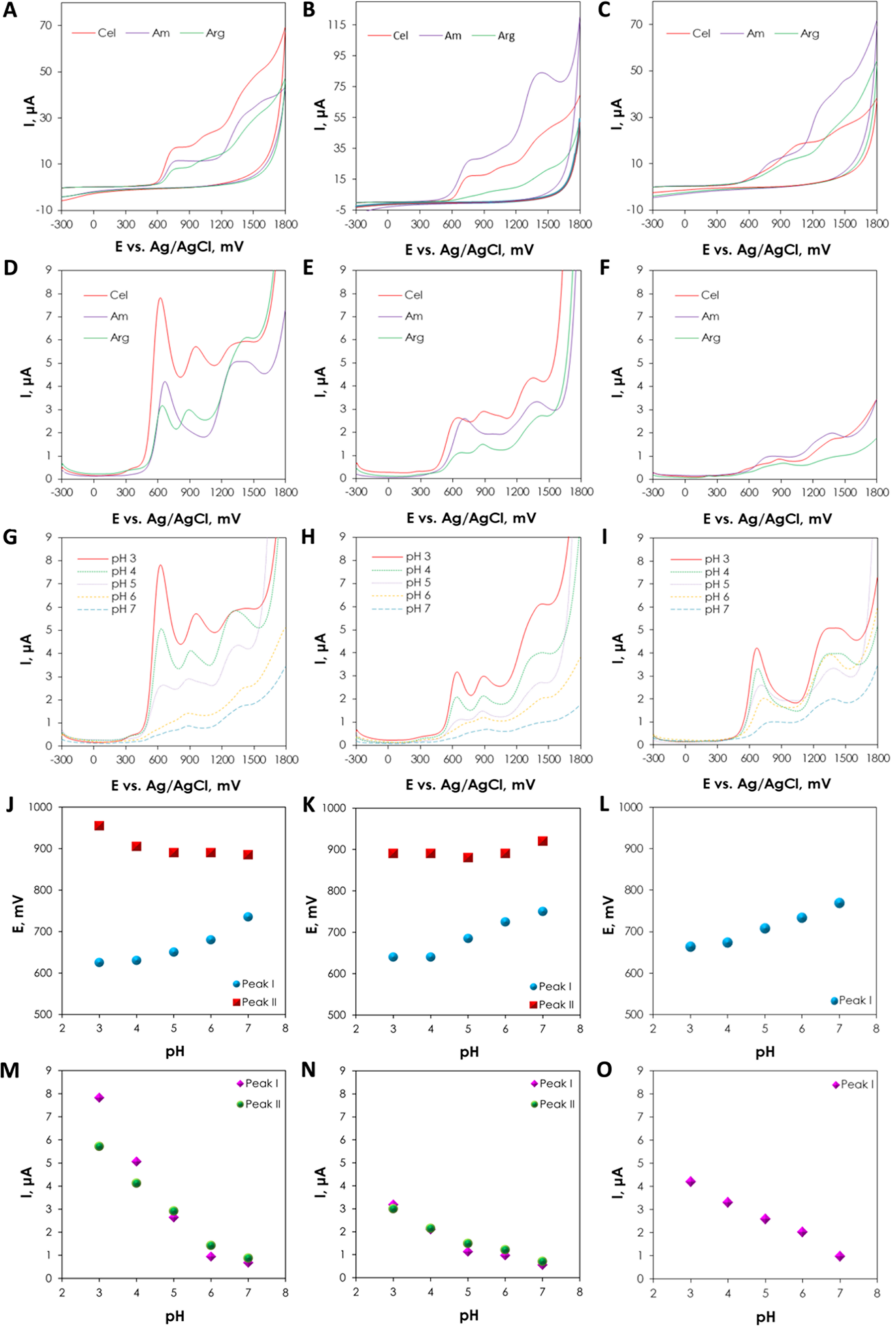
Cyclic voltammograms (A–C) and differential pulse voltammograms (D–I) registered for 1.4 mM celosianin, argentianin, and amaranthin in various buffers at pH 3 (A, D), pH 5 (B, E), and pH 7 (C, F), as well as the influence of pH on the anodic oxidation of Cel (G), Arg (H), and Am (I), respectively. pH dependence of the DPV oxidation potentials Epa^I^ and Epa^II^ for Cel (G, J), Arg (H, K), and Am (I, L), as well as the pH dependence of anodic current Ipa^I^ and Ipa^II^ Cel (M), Arg (N), and Am (O). CV voltammograms were recorded at a scan rate of 25 mV/s.

Cyclic voltammetry measurements provided general insights into the electroactivity of celosianin, argentianin, and amaranthin (Figure 4A-C). Cyclic voltammograms of 1.4 mM pigments were registered in 0.1 M acetate (pH 3–5) and phosphate (pH 6–7) buffers. Notably, celosianin and argentianin exhibited two peaks, while amaranthin showed only one peak. The anodic oxidation of amaranthin-type betacyanins occurs at the electroactive phenolic moiety (−OH) attached to carbon atom C-6, which is influenced by the glucuroglucosyl group linked to the glucose moiety at carbon atom C-5. The first peak likely corresponds to the phenolic hydroxyl group at carbon C-6, while the second peak may arise from the oxidation of ferulic and *p*-coumaric groups in celosianin and argentianin, respectively. Compared to betanidin,^32^ the oxidation potentials of acylated betalains were higher, indicating that betanidin exhibits stronger reduction properties due to the presence of the catecholic system in its structure.

#### 3.4.2 Differential Pulse Voltammetry Measurements

The pH dependence of the electrochemical oxidation of amaranthin **2**, argentianin **10**, and celosianin **17** was investigated by using the DPV method (Figure 4D–F). The oxidation potential of acylated betacyanins showed a shift toward more positive values with changes in pH (Figure 4G–I). Celosianin **17** exhibited a slightly lower oxidation potential than Arg and Am for peak I across the entire pH range. All of the pigments exhibited the lowest oxidation potential for the first peak at pH 4 (630 mV for Cel, 640 mV for Arg, and 675 mV for Am), and the oxidation potential increased in more alkaline media (pH 5–7). The oxidation potential of the second peak decreased within the entire pH range. For argentianin **10**, the minimum value was obtained at pH 5 (880 mV), and the anodic potential increased in alkaline pH 6–7 to 890 and 920 mV, respectively. However, for all pigments, there was a linear decrease in anodic current with increasing pH, with the highest anodic signals Ipa observed at pH 3 (ca. 7.83 μA for peak I and ca. 5.72 μA for peak II; Figure 4J–L). Additional results illustrating the plot of oxidation potential and anodic current as a function of pH are shown in Figure 4J–O. In more acidic media (pH 3–5), the voltammograms of celosianin and argentianin exhibit two distinct oxidation peaks (Figure 4G–I). As the pH becomes more alkaline (pH 6–7), the second peak becomes more prominent compared to the first, suggesting a different oxidation mechanism at higher and lower pH values (Figure 4G–I). This difference may be attributed to the higher stability of the oxidized betacyanin products at pH 5, resulting in slower chemical transformations. Betalains and their derivatives are known to be most stable at pH 5–5.5, leading to a higher concentration of oxidized forms and making them more available for subsequent reduction. The oxidation of the phenolic group proceeds irreversibly, generating a phenoxy radical, which is unstable and coexists in three resonant forms. The identification of short-lived intermediates of acylated betacyanins is challenging due to their reactivity and rapid rearrangement into more stable products. The oxidation reactions of acylated betacyanins may be accompanied by subsequent chemical processes, such as adsorption of the oxidation product or polymer formation at the electrode surface. Hydrolytic decomposition and polymerization of betalamic acid or *cyclo*-DOPA derivatives, resulting from the splitting of the aldimine bond, could be one of the irreversible transformations.^32^ The strong adsorption of oxidation products was confirmed by consecutive scans showing a decrease in the anodic current due to the surface blockage of the glassy carbon electrode at all pHs. The extensive chemical structures of acylated betalains prevent the direct calculation of thermodynamic parameters, e.g., formal oxidation potential *E*_o_ or electron stoichiometry, from the voltammograms. The voltammetry measurements proved that the oxidation of acylated betalain pigments is an irreversible, adsorption-controlled, and pH-dependent process, which adds complexity to the understanding of their electron transfer mechanism.

### 3.5 Antioxidant Activity of Oxidized Celosianins Compared to their Precursor, Celosianin

**Figure 5:**
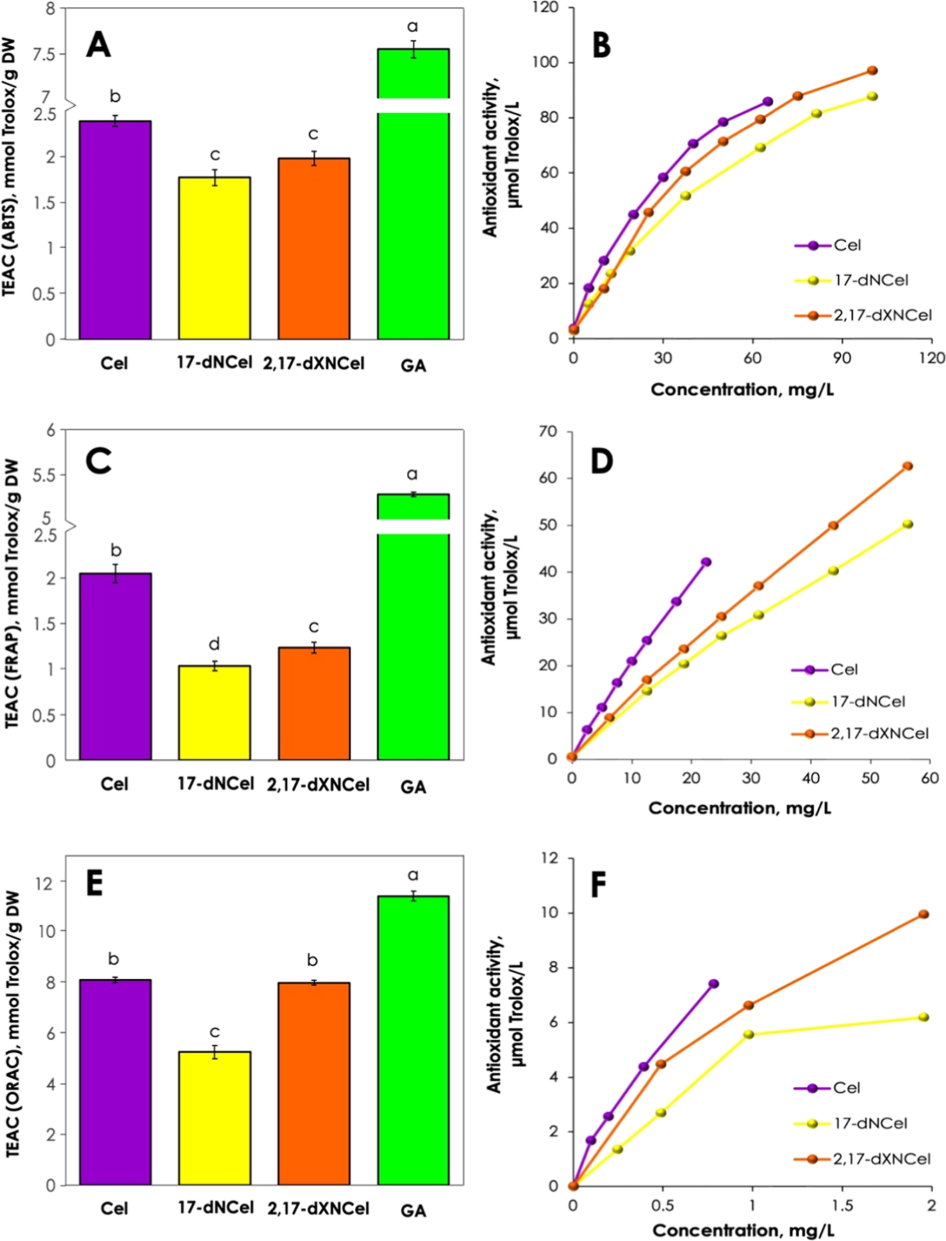
TEAC values determined for celosianin **17**, 17-decarboxy-neocelosianin **18**, 2,17-bidecarboxy-xanneocelosianin **20**, and gallic acid using ABTS (A), FRAP (C), and ORAC (E) assays as well as the dose-dependent antioxidant efficacy of the tested samples measured using ABTS (B), FRAP (D), and ORAC (F) assays. Raw data can be found in Supporting Table S2.

Phytochemicals with antioxidant properties have significant applications in food preservation, functional foods, and dietary supplements.^1^ The family of betalain pigments is known for its prominent antioxidant activity, comparable to well-known antioxidants like rutin, catechin, or ascorbic acid.^39^ However, to the best of our knowledge, previous studies have not investigated purified products of celosianin oxidation. Here, we evaluate the antioxidant activity of purified decarboxylated and dehydrogenated celosianins (17-decarboxy-neocelosianin **18** and 2,17-bidecarboxy-xanneocelosianin **20**) in comparison to the starting pigment (celosianin **17**). Common *in vitro* antioxidant assays such as ABTS, FRAP, and ORAC were employed to assess the radical scavenging activities of the purified oxidation products of celosianin. The results are presented in Figure 5 and Table S2. Compounds can react through different mechanisms, such as hydrogen atom transfer (HAT) or single electron transfer (SET). Their reactivity may vary based on pH, temperature, and reaction time. To adequately assess the antioxidant activity of compounds, multiple assays should be employed and the results compared. The ABTS assay measures the ability of a compound to scavenge ABTS cation radicals, indicating its antioxidant activity relative to Trolox. It involves both HAT and SET mechanisms, where radical quenching by hydrogen atom transfer or electron transfer occurs.^28^ In the FRAP assay, one electron is transferred to reduce Fe^3+^ into Fe^2+^ based on the SET mechanism of action.^29^ The ORAC assay measures the ability of an antioxidant to inhibit the oxidation reaction by a superoxide radical. It operates through a HAT mechanism, where the superoxide radical reacts with a fluorescent probe to form a nonfluorescent product. This test provides insight into the antioxidant processes that occur in the human body with characteristic conditions of 37 °C and pH 7.4.^30^

In the ABTS assay (Figure 5A), pure celosianin **17** exhibits a slightly higher TEAC (Trolox Equivalent Antioxidant Capacity) value (2.4 ± 0.06 mmol Trolox/g DW) compared to the oxidized derivatives, 17-decarboxy-neocelosianin **18** (1.8 ± 0.09 mmol Trolox/g DW) and 2,17-bidecarboxy-xanneocelosianin **20** (2.0 ± 0.08 mmol Trolox/g DW). This difference may be attributed to a lower number of active oxidation sites in 17-dNCel and 2,17-dXNCel due to prior dehydrogenation combined with decarboxylation. However, the antioxidant activity of celosianin is approximately 3 times lower than that of gallic acid, which is known for its potent radical scavenging properties, primarily due to the presence of 3 hydroxyl groups in its structure. These findings were also reflected in the IC_50_ assessment (Figure S8 and Table S2). The IC_50_ parameter represents the half-maximal inhibitory concentration of the pigment to reduce 50% of the radical cation present in the reaction mixture. Gallic acid displayed the lowest IC_50_ value (5.2 μg/mL), followed by celosianin (23 μg/mL), 2,17-dXNCel (29 μg/mL), and 17-dNCel (34 μg/mL).

Based on the FRAP assay (Figure 5C), celosianin **17** demonstrates nearly 2-fold higher antioxidant activity (2.0 ± 0.10 mmol Trolox/g DW) compared to 17-dNCel (1.0 ± 0.05 mmol Trolox/g DW) and 2,17-dXNCel (1.2 ± 0.06 mmol Trolox/g DW). Interestingly, the final oxidation product 2,17-bidecarboxy-xanneocelosianin **20** exhibits a slightly higher antioxidant activity than the first oxidation product 17-decarboxy-neocelosianin **18**.

In the ORAC assay (Figure 5E), no significant differences were observed between the antioxidant activities of 2,17-dXNCel (8.0 ± 0.10 mM TE/g DW) and celosianin (8.1 ± 0.15 mM TE/g DW). This could be attributed to the degradation of thermolabile celosianin during the half-hour incubation and 1 h reaction at 37 °C, resulting in the formation of decarboxylated and dehydrogenated derivatives. It has been proven previously that the stability of betalains decreases with the degree of purification, rendering celosianin more susceptible to degradation/oxidation. It is important to note that the applied temperature in the ORAC assay is similar to conditions found in the human body, where partial decomposition of pigments can occur. The antioxidant activity of 17-dNCel was determined to be 5.9 ± 0.26 mmol Trolox/g DW, showing that it can potentially be less active than Cel and 2,17-dXNCel.

All of the tested samples also demonstrated a concentration-dependent increase in radical scavenging assays (Figure 5B,D,F), with their antioxidant activity increasing with higher compound concentrations. At low concentrations, the changes in antioxidant activity were linear, but as concentrations increased, the slope of the curves decreased. In the case of the ABTS assay, this can be attributed to hindered steric access to ABTS^+•^ due to the complex structure of the betacyanins. The reaction between pigments and ABTS^+•^ may become more challenging, resulting in a slower reaction rate at higher pigment concentrations.

### 3.6 Correlations between TEAC Values of Compounds in ABTS, FRAP, and ORAC Assays

The results of the ABTS and FRAP assays showed a strong linear relationship, as evidenced by the high Pearson correlation coefficient of 0.99. This may result from the same SET mechanism, which is involved in both assays.

The lower correlation coefficient value (*r* = 0.88) between the FRAP and ORAC test results, compared to the ABTS – FRAP assays, suggests the presence of a different dominant mechanism of action and the influence of reaction conditions. The ORAC test is performed at pH 7.4, which is less favorable for both the oxidation reactions and the stability of celosianin and its oxidation derivatives. Additionally, the different reaction times (10 min for FRAP and 1 h for ORAC) may contribute to the observed differences. It should be noted that the FRAP test, which measures the reduction capacity of compounds based on iron ions, does not directly reflect the effect of the tested samples on the physiological system. However, this comparison highlights the capability of betacyanin pigments to undergo oxidation reactions through both the SET and HAT mechanisms.

A positive correlation was also observed between the results of the ORAC and ABTS tests (*r* = 0.91). Although both tests utilize radicals as oxidants, the compounds used in each test exhibit a distinct specificity. The ORAC test involves the use of superoxide radicals, which are small and play a role in the body’s defense mechanisms. In contrast, ABTS cation radicals do not naturally occur in mammals and serve as nonphysiological source. The dominant mechanism in the ABTS test is typically SET, although the HAT mechanism is also possible. Conversely, the ORAC test exclusively employs the HAT mechanism. In addition, in the ORAC test, compound decomposition may occur due to the reaction temperature.

### 3.7 Biological Activity of Oxidized Derivatives of Celosianin

The role of nutrition as a vital component of a healthy lifestyle and its benefits in supporting patients during therapy and recovery have gained recognition. Cardiotoxicity, a serious medical complication that occurs during anticancer therapy, adversely affects the quality of patient lives. However, phytochemicals derived from dietary sources, such as fruits and vegetables, have been identified as evidence-based compounds with cardioprotective properties. Phytochemicals have the potential to alleviate side effects experienced during adjuvant and targeted therapy.^40^ In our previous report,^2^ we demonstrated that betalain-rich *A. hortensis* var. *rubra* extracts and purified pigments enhance the resistance of rat cardiomyocytes to H_2_O_2_-induced cell damage and elevate glutathione levels in H9c2 cells. In this study, we evaluated the cardioprotective activity of oxidized forms of celosianin (17-dNCel and 2,17-dXNCel) in H9c2 cardiomyocyte cells for the first time.

#### 3.7.1 Cytotoxic Activity of Oxidized Forms of Celosianin

**Figure 6:**
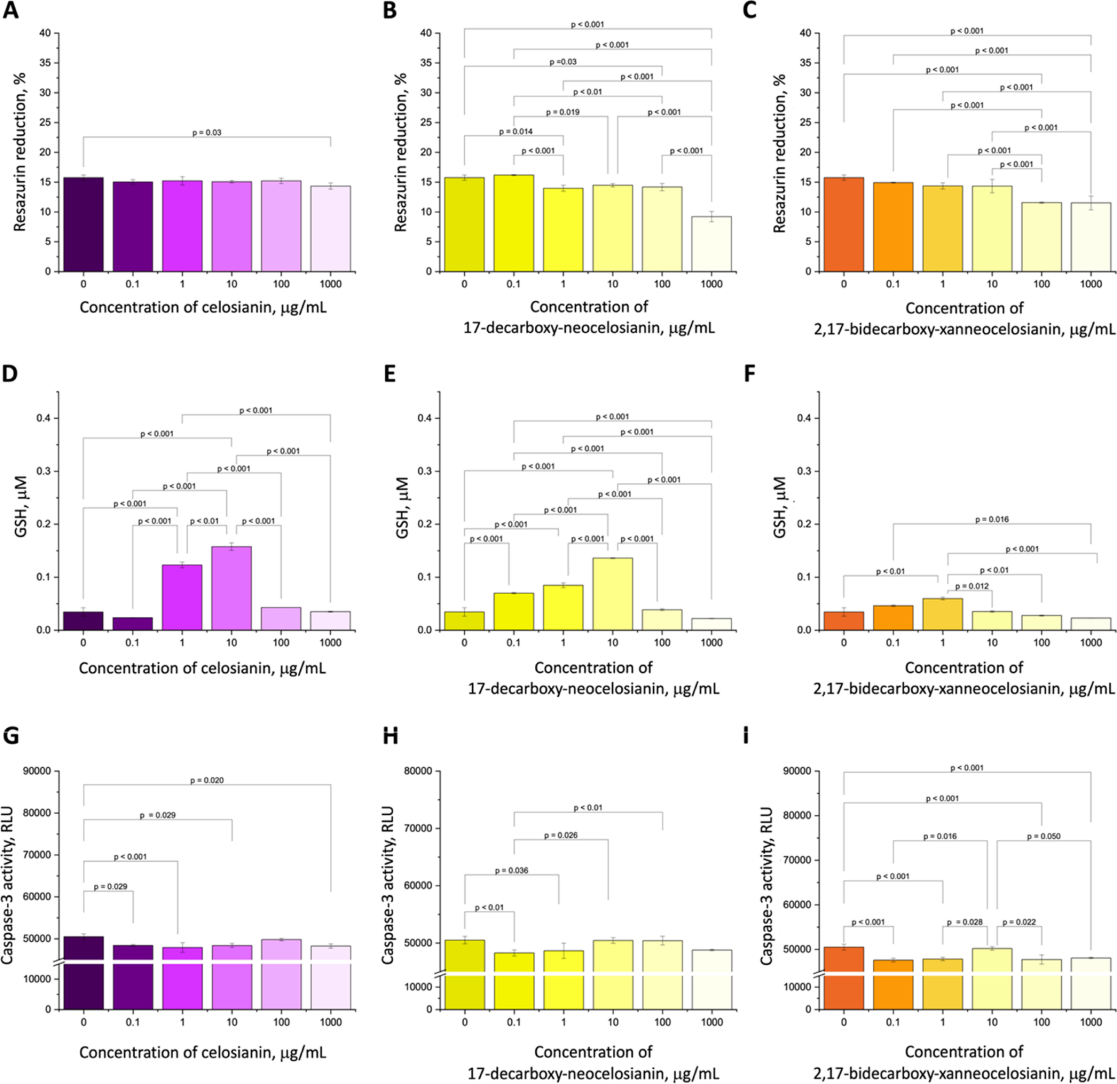
Metabolic activity of H9c2 cells incubated with varying concentrations of celosianin (A), 17-decarboxy-neocelosianin (B), and 2,17-bidecarboxy-xanneocelosianin (C) compared to the control (0 μg/mL)—cells cultured in a medium without tested betalains. H9c2 cell response to H_2_O_2_-induced damage (D–F) and PAC-induced apoptosis (G–I) after treatment with different concentrations of celosianin (D, G), 17-decarboxy-neocelosianin (E, H), and 2,17-bidecarboxy-xanneocelosianin (F, I) compared to the control (0 μg/mL)—cells cultured in a medium without tested betalains but supplemented with 500 μM H_2_O_2_ (D–F) or 80 nM PAC (G–I).

Our results show that the tested compounds were well-tolerated by H9c2 cells with no cytotoxic response observed in a wide concentration range, as determined by the alamarBlue resazurin reduction assay. The control samples (0 μg/mL betalains) showed a 15.8% reduction in resazurin. At the highest concentration of pigments (1 mg/mL), it resulted in approximately 42 and 23% decrease in cell viability, respectively, compared to the control cells (Figure 6B–C). In contrast, celosianin **17** showed no toxicity even at the highest concentration (Figure 6A), consistent with previous reports on celosianin viability.^2^ To confirm these findings, we performed fluorescent live/dead staining and observed that H9c2 cells treated with Cel, 17-dNCel, and 2,17-dXNCel exhibited normal cell morphology with a characteristic spindle shape similar to that of the control (Figure S9).

#### 3.7.2 Cardioprotective Activity of Oxidized Celosianins in H_2_O_2_- and PAC-Induced Model of H9c2 Cells

The safety profile of the tested samples was evaluated to assess the influence of celosianin **17** derivatives in preventing cardiomyocyte damage (Figure 6D–F) and apoptosis (Figure 6G–I) in a rat cardiomyocyte model injured by H_2_O_2_ and Paclitaxel (PAC) in concentrations ranging from 0.1 to 1000 μg/mL. To investigate whether the pigments could prevent cells from reactive oxygen species (ROS)-induced damage, H9c2 myoblasts were preincubated with celosianin **17**, 17-decarboxy-neocelosianin **18**, and 2,17-bidecarboxy-xanneocelosianin **20** for 24 h before adding 500 μM H_2_O_2_ to induce oxidative damage. After 24 h, the level of glutathione (GSH) was measured. As in the case of results obtained previously for *A. hortensis* extracts and purified pigments,^2^ a biphasic dose response known as the hormesis phenomenon was observed (Figure 6D–F), with a low-dose stimulation and high-dose inhibition. All analyzed compounds exerted a dose-dependent protective activity against H_2_O_2_-induced oxidative damage up to 10 μg/mL for Cel and 17-dNCel and up to 1 μg/mL for 2,17-dXNCel. Celosianin showed the highest increase in the GSH level (4.5-fold increase compared to control), followed by 17-decarboxy-neocelosianin (4-fold increase) and 2,17-bidecarboxy-xanneocelosianin (1.7-fold increase).

To further understand the protective effects of pigments against apoptosis, a major form of programmed cell death, we cultured H9c2 cells in the presence of Cel, 17-dNCel, and 2,17-dXNCel. After 24 h of incubation, an apoptosis inducer (PAC, 80 nM) was introduced. PAC is a highly potent chemotherapy agent that can induce side effects in patients, including cardiovascular issues. The results show that the studied samples provided only limited protection against PAC-induced cell death (Figure 6G–I). The presence of Cel, 17-dNCel, and 2,17-dXNCel resulted in a decrease in caspase-3 activity of approximately 0.1–6% compared to the control (cells only, no pigments added).

Based on our preliminary findings, the decarboxylated and dehydrogenated forms of celosianin support the hypothesis that betalain pigments are unlikely to have a negative impact on the human body following administration and digestion. However, it is important to note that *in vitro* studies conducted on cell lines do not accurately reflect the results obtained *in vivo* using animal models. Therefore, more extensive research is required to gain a comprehensive understanding of the biological effects of betalains.

## Supporting Information Available

Mass spectrometric chromatograms of celosianin and its oxidation products registered at different time points of the reaction (Figure S1); high-resolution mass spectrometric data (LC-Q-Orbitrap-MS) obtained for amaranthin, argentianin, celosianin, and their oxidation products (Table S1); ^1^H NMR (Figures S2, S4, S6) and ^13^C NMR (Figure S3, S5, S7) spectra registered for 17-decarboxy-neocelosianin, 2-decarboxy-xanneocelosianin, and 2,17-bidercarboxy-xanneocelosianin, respectively, the ABTS, FRAP, and ORAC antioxidant activity results obtained for celosianin and its oxidation products (Table S2); IC_50_ values calculated for celosianin and oxidized celosianins based on the ABTS assay (Figure S8); and fluorescence live/dead imaging of H9c2 cells pretreated with celosianins and its oxidized derivatives compared to control (Figure S9) (PDF)
