# Supplementary material for: Unveiling Alternative Oxidation Pathways and Antioxidant and Cardioprotective Potential of Amaranthin-Type Betacyanins from Spinach-like Atriplex hortensis var. ‘Rubra’
Source: J Agric Food Chem. 2023 Oct 4;71(41):15017–34. doi: 10.1021/acs.jafc.3c03044 (PMC10591473; doi:10.1021/acs.jafc.3c03044)
Supplement: Supplementary file 1 — jf3c03044_si_001.pdf [file jf3c03044_si_001.pdf]

Supporting information

Unveiling Alternative Oxidation Pathways and Antioxidant and Cardioprotective Potential of Amaranthin-Type Betacyanins from Spinach-like *Atriplex hortensis* var. '*Rubra*'

Agnieszka Kumorkiewicz-Jamro[a,b,c*], Renata Górska[a], Małgorzata Krok-Borkowicz[d], Przemysław Mielczarek[e,f], Łukasz Popenda[g], Kateryna Lystvan[h], Elżbieta Pamuła[d], Sławomir Wybraniec[a]

[a] Department of Chemical Technology and Environmental Analysis, Faculty of Chemical Engineering and Technology, Cracow University of Technology, Warszawska 24, 31-155 Cracow, Poland

[b] South Australian Health and Medical Research Institute, Adelaide 5000 SA, Australia

[c] Faculty of Health and Medical Sciences, University of Adelaide, Adelaide 5005 SA, Australia

[d] Department of Biomaterials and Composites, Faculty of Materials Science and Ceramics, AGH University of Science and Technology, Al. Mickiewicza 30, 30-059 Cracow, Poland

[e] Department of Analytical Chemistry and Biochemistry, Faculty of Materials Science and Ceramics, AGH University of Science and Technology, Al. Mickiewicza 30, 30059, Cracow, Poland

[f] Laboratory of Proteomics and Mass Spectrometry, Maj Institute of Pharmacology, Polish Academy of Sciences, Smętna 12, 31-343 Cracow, Poland

[g] NanoBioMedical Centre, Adam Mickiewicz University, Wszechnicy Piastowskiej 3, 61-614 Poznań, Poland

[h] Department of Genetic Engineering, Institute of Cell Biology and Genetic Engineering of National Academy of Sciences of Ukraine (NASU), Academika Zabolotnoho, 148, 03143, Kyiv, Ukraine

* Corresponding author:
Agnieszka Kumorkiewicz-Jamro
agnieszka.kumorkiewicz-jamro@pk.edu.pl
ORCID: 0000-0002-3828-3128

Results

Mass spectrometric chromatograms depicting the main products of the oxidation of purified celosianin by 1 mM ABTS radicals. The reaction was carried out in the presence of an acetate buffer at pH 3. Chromatograms were registered after 5, 20, 40, 60, and 80 min of reaction time. Chromatographic separation was conducted using a 150 mm x 4.6 mm, 5.0 µm Kinetex C_{18} column (Phenomenex, Torrance, CA, USA). The mobile phase was composed on 2% aqueous formic acid (eluent A) and methanol (eluent B), with a gradient elution as follows: (t [min], %B), (0, 10), (12, 40), (15, 80). Data were recorded in positive ion polarity using selected ion monitoring (SIM). The chemical structures of oxidized celosianins, namely: 17-decarboxy-neocelosianin (*m/z* 857) and 2,17-bidecarboxyxanneocelosianin (*m/z* 811), were confirmed through LC-Q-Orbitrap-MS (Table S1) and NMR analyses (Figures S2-S7).

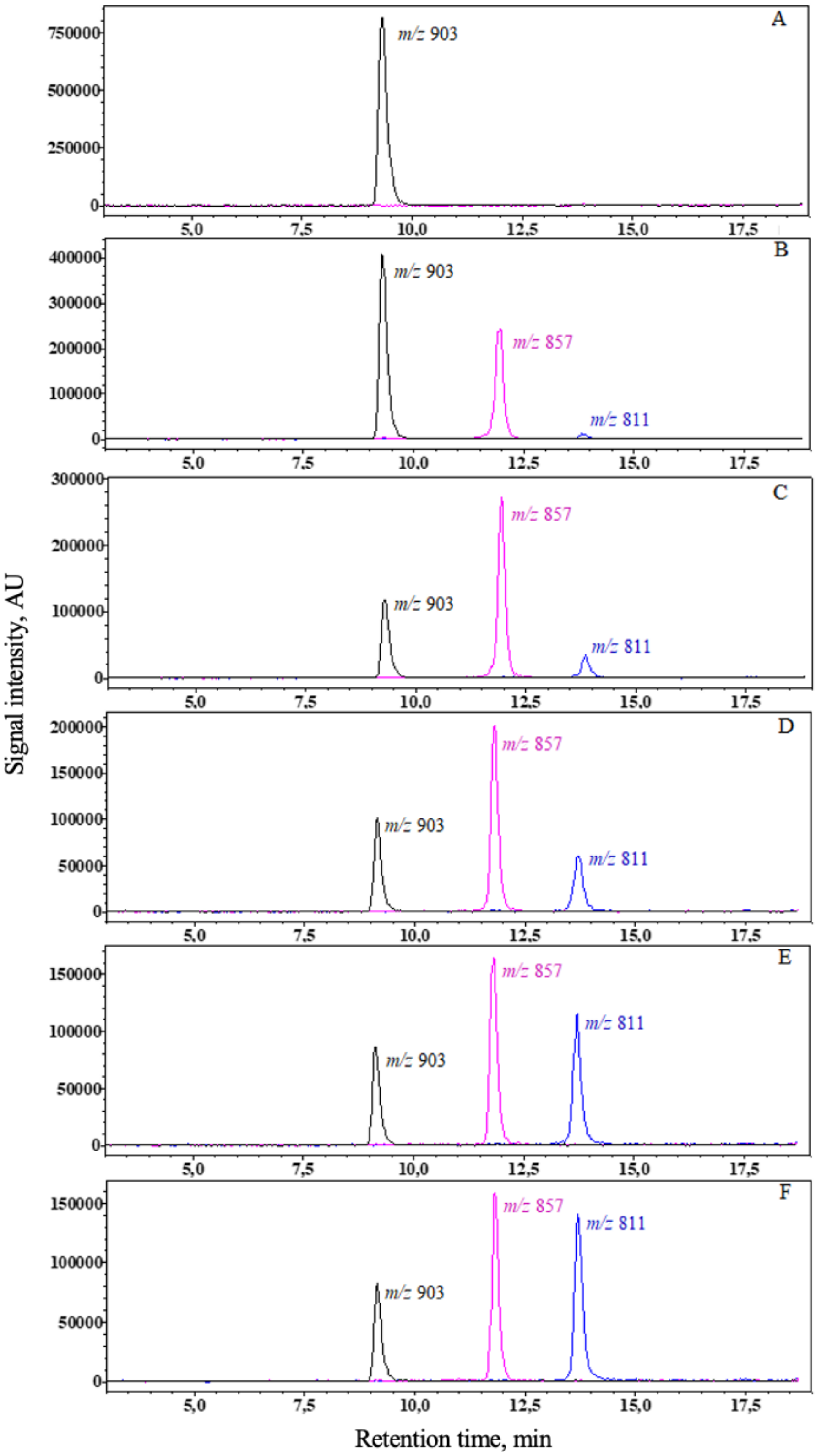

Figure S1 LC-MS chromatograms in SIM mode of celosianin (A) and the main products formed during celosianin oxidation by ABTS radicals at pH 3 registered after 5 (B); 20 (C); 40 (D); 60 (E); and 80 (F) min of reaction.

High-resolution mass spectrometric (HRMS) data obtained using LC-Q-Orbitrap-MS

The molecular formulas and the fragmentation patterns of the oxidation products of amaranthin, argentianin, and celosianin were determined through HRMS analyses using the product ion mode, where targeted precursors were isolated and fragmented in the HCD cell. The fragmentation ions (MS^2) were analyzed in the Orbitrap analyzer of the Orbitrap Exploris™ 240 Mass Spectrometer. Based on the obtained results and the elucidation of chemical structures by NMR, a possible mechanism for the oxidation of amaranthin-type betacyanins was proposed.

Table S1 High-resolution mass spectrometric data obtained using LC-Q-Orbitrap-MS system for oxidized betacyanins as well as for their fragmentation ions.

**Pigments and fragmentation ions[a]**	**$[M+H]^+$ molecular formula**	**$[M+H]^+$ observed**	**$[M+H]^+$ predicted**	**Error [mDa]**	**Error [ppm]**
		Amaranthin oxidation			
2,3-dihydroxy-2-decarboxy-xanamaranthin **1**	$C_{29}H_{35}N_2O_{19}$	715.1831	715.1829	0.2	0.28
nl: - Gluc	$C_{23}H_{27}N_2O_{13}$	539.1512	539.1508	0.4	0.74
nl: - Gluc/Glc	$C_{17}H_{17}N_2O_8$	377.0978	377.0979	-0.1	-0.27
nl: - H_2O/Gluc/Glc	$C_{17}H_{15}N_2O_7$	359.0877	359.0874	0.3	0.84
amaranthin **2**	$C_{30}H_{35}N_2O_{19}$	727.1830	727.1829	0.1	0.14
nl: -Gluc	$C_{24}H_{27}N_2O_{13}$	551.1517	551.1508	0.9	1.63
nl: -Gluc/Glc	$C_{18}H_{17}N_2O_8$	389.0981	389.0979	0.2	0.51
nl: -Gluc/Glc/ CO_2/2H	$C_{17}H_{15}N_2O_6$	343.0926	343.0925	0.1	0.29
nl: -Gluc/Glc/ 2CO_2/4H	$C_{16}H_{13}N_2O_4$	297.0872	297.0870	0.2	0.67
nl: -Gluc/Glc/ 3CO_2/2H	$C_{15}H_{15}N_2O_2$	255.1127	255.1128	-0.1	-0.39
2-decarboxy-xanamaranthin **3**	$C_{29}H_{33}N_2O_{17}$	681.1771	681.1774	-0.3	-0.44
nl: -Gluc	$C_{23}H_{25}N_2O_{11}$	505.1452	505.1453	-0.1	-0.20
nl: -Gluc/CO_2/2H	$C_{22}H_{23}N_2O_9$	459.1395	459.1398	-0.3	-0.65
nl: -Gluc/Glc	$C_{17}H_{15}N_2O_6$	343.0923	343.0925	-0.2	-0.58
nl: -Gluc/Glc/CO_2/2H	$C_{16}H_{13}N_2O_4$	297.0869	297.0870	-0.1	-0.34
nl: -Gluc/Glc/2CO_2	$C_{15}H_{15}N_2O_2$	255.1125	255.1128	-0.3	-1.18
2,17-bidecarboxy-xanamaranthin **4**	$C_{28}H_{33}N_2O_{15}$	637.1876	637.1876	0.0	0.00
nl: -Gluc	$C_{22}H_{25}N_2O_9$	461.1557	461.1555	0.2	0.43
nl: -Gluc/CO_2	$C_{21}H_{25}N_2O_7$	417.1653	417.1656	-0.3	-0.72
nl: -Gluc/Glc	$C_{16}H_{15}N_2O_4$	299.1027	299.1026	0.1	0.33
nl: -Gluc/Glc/CO_2/2H	$C_{15}H_{13}N_2O_2$	253.0972	253.0972	0.0	0.00
17-decarboxy-neoamaranthin **5**	$C_{29}H_{33}N_2O_{17}$	681.1772	681.1774	-0.2	-0.29
nl: -Gluc	$C_{23}H_{25}N_2O_{11}$	505.1451	505.1453	-0.2	-0.40

nl: -Gluc/CO_2/2H	$C_{22}H_{23}N_2O_9$	459.1392	459.1398	-0.6	-1.31
nl: -Gluc/Glc	$C_{17}H_{15}N_2O_6$	343.0921	343.0925	-0.4	-1.17
nl: -Gluc/Glc/CO_2/2H	$C_{16}H_{13}N_2O_4$	297.0867	297.0870	-0.3	-1.01
nl: -Gluc/Glc/2CO_2	$C_{15}H_{15}N_2O_2$	255.1124	255.1128	-0.4	-1.57
2,17-bidecarboxy-xanneoamaranthin **6**	$C_{28}H_{31}N_2O_{15}$	635.1719	635.1719	0.0	0.00
nl: -Gluc	$C_{22}H_{23}N_2O_9$	459.1403	459.1398	0.5	1.09
nl: -Gluc/Glc	$C_{16}H_{13}N_2O_4$	297.0871	297.0870	0.1	0.34
2,17-decarboxy-neoamaranthin **7**	$C_{28}H_{33}N_2O_{15}$	637.1879	637.1875	0.4	0.63
nl: -Gluc	$C_{22}H_{25}N_2O_9$	461.1556	461.1555	0.1	0.22
nl: -Gluc/Glc	$C_{16}H_{15}N_2O_4$	299.1027	299.1026	0.1	0.33
nl: -Gluc/Glc/CO_2	$C_{15}H_{15}N_2O_2$	255.1127	255.1128	-0.1	-0.39
nl: -Gluc/Glc/CO_2/2H	$C_{15}H_{13}N_2O_2$	253.0972	253.0971	0.1	0.40
2-decarboxy-xanneoamaranthin **8**	$C_{29}H_{31}N_2O_{17}$	679.1619	679.1617	0.2	0.29
nl: -Gluc	$C_{23}H_{23}N_2O_{11}$	503.1297	503.1296	0.1	0.20
nl: - Gluc/Glc	$C_{17}H_{13}N_2O_6$	341.0769	341.0786	-1.7	-4.98
nl: - Gluc/Glc/2CO_2	$C_{15}H_{13}N_2O_2$	253.0978	253.0972	0.6	2.37
		Argentianin oxidation			
argentianin **10**	$C_{39}H_{41}N_2O_{21}$	873.2198	873.2196	0.2	0.23
nl: -Coum/Gluc	$C_{24}H_{27}N_2O_{13}$	551.1509	551.1508	0.1	0.24
nl: -Coum/Gluc/Glc	$C_{18}H_{17}N_2O_8$	389.0981	389.0979	0.2	0.41
nl: -Coum/Gluc/Glc/ CO_2/2H	$C_{17}H_{15}N_2O_6$	343.0928	343.0925	0.3	0.99
nl: -Coum/Gluc/Glc/ 2CO_2/4H	$C_{16}H_{13}N_2O_4$	297.0872	297.0870	0.2	0.74
nl: -Coum/Gluc/Glc/ 3CO_2/2H	$C_{15}H_{15}N_2O_2$	255.1131	255.1128	0.3	1.18
2,3-dihydroxy-2-decarboxy-xanargentianin **9**	$C_{38}H_{41}N_2O_{21}$	861.2198	861.2196	0.2	0.23
nl: - Coum/Gluc	$C_{23}H_{27}N_2O_{13}$	539.1512	539.1508	0.4	0.74
nl: - Coum/Gluc/Glc	$C_{17}H_{17}N_2O_8$	377.0977	377.0979	-0.2	-0.53
nl: - H_2O/Coum/Gluc/Glc	$C_{17}H_{15}N_2O_7$	359.0872	359.0874	-0.2	-0.56
17-decarboxy-neoargentianin **11**	$C_{38}H_{39}N_2O_{19}$	827.2122	827.2142	-2.0	-2.42
nl: -Coum	$C_{29}H_{33}N_2O_{17}$	681.1788	681.1774	1.4	2.10
nl: -Coum/Gluc	$C_{23}H_{25}N_2O_{11}$	505.1461	505.1453	0.8	1.60
nl: -Coum/Gluc/Glc	$C_{17}H_{15}N_2O_6$	343.0927	343.0925	0.2	0.70
nl: -Coum/Gluc/CO_2/2H	$C_{16}H_{13}N_2O_4$	297.0872	297.0870	0.2	0.74
2,17-decarboxy-xanargentianin **12**	$C_{37}H_{39}N_2O_{17}$	783.2240	783.2243	-0.3	-0.38
nl: -Coum	$C_{28}H_{33}N_2O_{15}$	637.1873	637.1875	-0.2	-0.31
nl: -Coum/Gluc	$C_{22}H_{25}N_2O_9$	461.1555	461.1555	0.0	0.00
nl: -Coum/Gluc/Glc	$C_{16}H_{15}N_2O_4$	299.1027	299.1026	0.1	0.33
nl: -Coum/Gluc/Glc/CO_2	$C_{15}H_{15}N_2O_2$	255.1127	255.1128	-0.1	-0.39
nl: -Coum/Gluc/Glc/CO_2/2H	$C_{15}H_{13}N_2O_2$	253.0973	253.0971	0.2	0.79
2,17-bidecarboxy-xanneoargentianin **13**	$C_{37}H_{37}N_2O_{17}$	781.2090	781.2087	0.3	0.42
nl: -Coum/Gluc	$C_{22}H_{23}N_2O_9$	459.1391	459.1398	-0.7	-1.55

nl: -Coum/Gluc/Glc	$C_{16}H_{13}N_2O_4$	297.0871	297.0870	0.1	0.34
nl: -Coum/Gluc/Glu/CO_2	$C_{15}H_{13}N_2O_2$	253.0973	253.0972	0.1	0.40
2,17-bidecarboxy-neoargentianin **14**	$C_{37}H_{39}N_2O_{17}$	783.2240	783.2243	-0.3	-0.41
nl: -Coum	$C_{28}H_{33}N_2O_{15}$	637.1873	637.1876	-0.2	-0.39
nl: -Coum/Gluc	$C_{22}H_{25}N_2O_9$	461.1555	461.1555	0.0	0.09
nl: -Coum/Gluc/CO_2	$C_{21}H_{25}N_2O_7$	417.1652	417.1656	-0.4	-1.03
nl: -Coum/Gluc/Glc	$C_{16}H_{15}N_2O_4$	299.1027	299.1026	0.1	0.23
nl: -Coum/Gluc/Glc/CO_2/2H	$C_{15}H_{13}N_2O_2$	253.0972	253.0972	0.0	0.0
2-decarboxy-xanneoargentianin **15**	$C_{38}H_{37}N_2O_{19}$	825.1988	825.1985	0.3	0.36
nl: -Coum	$C_{29}H_{31}N_2O_{17}$	679.1622	679.1617	0.5	0.74
nl: -Coum/Gluc	$C_{23}H_{23}N_2O_{11}$	503.1297	503.1296	0.1	0.20
nl: -Coum/Gluc/Glc	$C_{17}H_{13}N_2O_6$	341.0783	341.0786	-0.3	-0.88
nl: -Coum/Gluc/Glc/CO_2	$C_{16}H_{13}N_2O_4$	297.0876	297.0870	0.6	2.02
	Celosianin oxidation				
celosianin **17**	$C_{40}H_{43}N_2O_{22}$	903.2306	903.2302	0.4	0.44
nl: -Fer/Gluc	$C_{24}H_{27}N_2O_{13}$	551.1523	551.1508	1.5	2.72
nl: -Fer/Gluc/Glc	$C_{18}H_{17}N_2O_8$	389.0983	389.0979	0.4	1.03
nl: -Fer/Gluc/Glc/CO_2/2H	$C_{17}H_{15}N_2O_6$	343.0929	343.0925	0.4	1.17
nl: -Fer/Gluc/Glc/2CO_2/4H	$C_{16}H_{13}N_2O_4$	297.0874	297.0870	0.4	1.35
nl: -Fer/Gluc/Glc/3CO_2/4H	$C_{15}H_{13}N_2O_2$	253.0974	253.0972	0.2	0.79
2,3-dihydroxy-2-decarboxy-xancelosianin **16**	$C_{39}H_{43}N_2O_{22}$	891.2305	891.2302	0.3	0.34
nl: - H_2O	$C_{39}H_{41}N_2O_{21}$	873.2192	873.2196	-0.4	-0.46
nl: - H_2O/CO_2	$C_{38}H_{41}N_2O_{19}$	829.2292	829.2298	-0.6	-0.72
nl: - Fer/Gluc	$C_{23}H_{27}N_2O_{13}$	539.1511	539.1508	0.3	0.56
nl: - H2O/Fer/Gluc	$C_{23}H_{25}N_2O_{12}$	521.1396	521.1402	-0.6	-1.15
nl: - Fer/Gluc/Glc	$C_{17}H_{17}N_2O_8$	377.0978	377.0979	-0.1	-0.27
nl: - H_2O/Fer/Gluc/Glc	$C_{17}H_{15}N_2O_7$	359.0875	359.0874	0.1	0.28
17-decarboxy-neocelosianin **18**	$C_{39}H_{41}N_2O_{20}$	857.2237	857.2247	-1.0	-1.17
nl: -Fer	$C_{29}H_{33}N_2O_{17}$	681.1777	681.1774	0.3	0.44
nl: -Fer/Gluc	$C_{23}H_{25}N_2O_{11}$	505.1457	505.1453	0.4	0.79
nl: -Fer/Gluc/CO_2/2H	$C_{22}H_{23}N_2O_9$	459.1414	459.1398	1.6	3.48
nl: -Fer/Gluc/Glc	$C_{17}H_{15}N_2O_6$	343.0926	343.0925	0.1	0.29
nl: -Fer/Gluc/Glc/CO_2/2H	$C_{16}H_{13}N_2O_4$	297.0872	297.0870	0.2	0.67
nl: -Fer/Gluc/Glc/2CO_2/2H	$C_{15}H_{13}N_2O_2$	253.0974	253.0972	0.2	0.79
2,17-bidecarboxy-xancelosianin **19**	$C_{38}H_{41}N_2O_{18}$	813.2353	813.2349	0.4	0.49
nl: -Fer/Gluc	$C_{22}H_{25}N_2O_9$	461.1556	461.1555	0.1	0.22
nl: -Fer/Gluc/Glc	$C_{21}H_{25}N_2O_7$	299.1027	299.1026	0.1	0.33
nl: -Fer/Gluc/Glc/CO_2	$C_{16}H_{15}N_2O_4$	255.1129	255.1128	0.1	0.39
nl: -Fer/Gluc/Glc/CO_2/2H	$C_{15}H_{13}N_2O_2$	253.0970	253.0971	-0.1	-0.40
2,17-bidecarboxy-xanneocelosianin **20**	$C_{38}H_{39}N_2O_{18}$	811.2197	811.2192	0.5	0.62

nl: -Fer/Gluc	$C_{22}H_{23}N_2O_9$	459.1402	459.1398	0.4	0.87
nl: -Fer/Gluc/Glc	$C_{16}H_{13}N_2O_4$	297.0872	297.0870	0.2	0.67
nl: -Fer/Gluc/Glc/CO_2	$C_{15}H_{13}N_2O_2$	253.0972	253.0972	0.0	0.00
2,17-bidecarboxy-neocelosianin **21**	$C_{38}H_{41}N_2O_{18}$	813.2353	813.2349	0.4	0.49
nl: -Fer/Gluc	$C_{22}H_{25}N_2O_9$	461.1554	461.1556	-0.2	-0.43
nl: -Fer/Gluc/CO_2	$C_{21}H_{25}N_2O_7$	417.1651	417.1656	-0.5	-1.20
nl: -Fer/Gluc/Glc	$C_{16}H_{15}N_2O_4$	299.1027	299.1026	0.1	0.33
nl: -Fer/Gluc/Glc/CO_2/2H	$C_{15}H_{13}N_2O_2$	253.0973	253.0972	0.1	0.40
2-decarboxy-xanneocelosianin **22**	$C_{39}H_{39}N_2O_{20}$	855.2088	855.2091	-0.3	-0.35
nl: -Fer/Gluc	$C_{23}H_{23}N_2O_{11}$	503.1301	503.1296	0.5	0.99
nl: -Fer/Gluc/CO_2	$C_{22}H_{23}N_2O_9$	459.1399	459.1398	0.1	0.22
nl: -Fer/Gluc/Glc	$C_{17}H_{13}N_2O_6$	341.0769	341.0768	0.1	0.29
nl: -Fer/Gluc/Glc/CO_2/2H	$C_{16}H_{11}N_2O_4$	295.0714	295.0715	-0.1	-0.34

[a] nl – neutral losses from *[M + H]$^+$; Gluc* – glucuronosyl, Glc – glucosyl, Coum – coumaroyl, Fer – feruloyl

^{1}H NMR and ^{13}C NMR spectra for the oxidized celosianins

The structure elucidation of 2,17-dXNCel and 2-dXNCel was performed in DMSO-*d6*/TFA-*d*, while that for 17-dNCel was carried out in D_2O. Due to the non-destructive effect of D_2O, it was chosen as the solvent for prolong NMR measurements of 17-decarboxyneocelosianin. All spectra were acquired using an Agilent DD2 800 (18.8 T) spectrometer (Agilent Technologies, Santa Clara, CA, USA). Chemical shifts were determined relative to the internal TMSP-d_4.

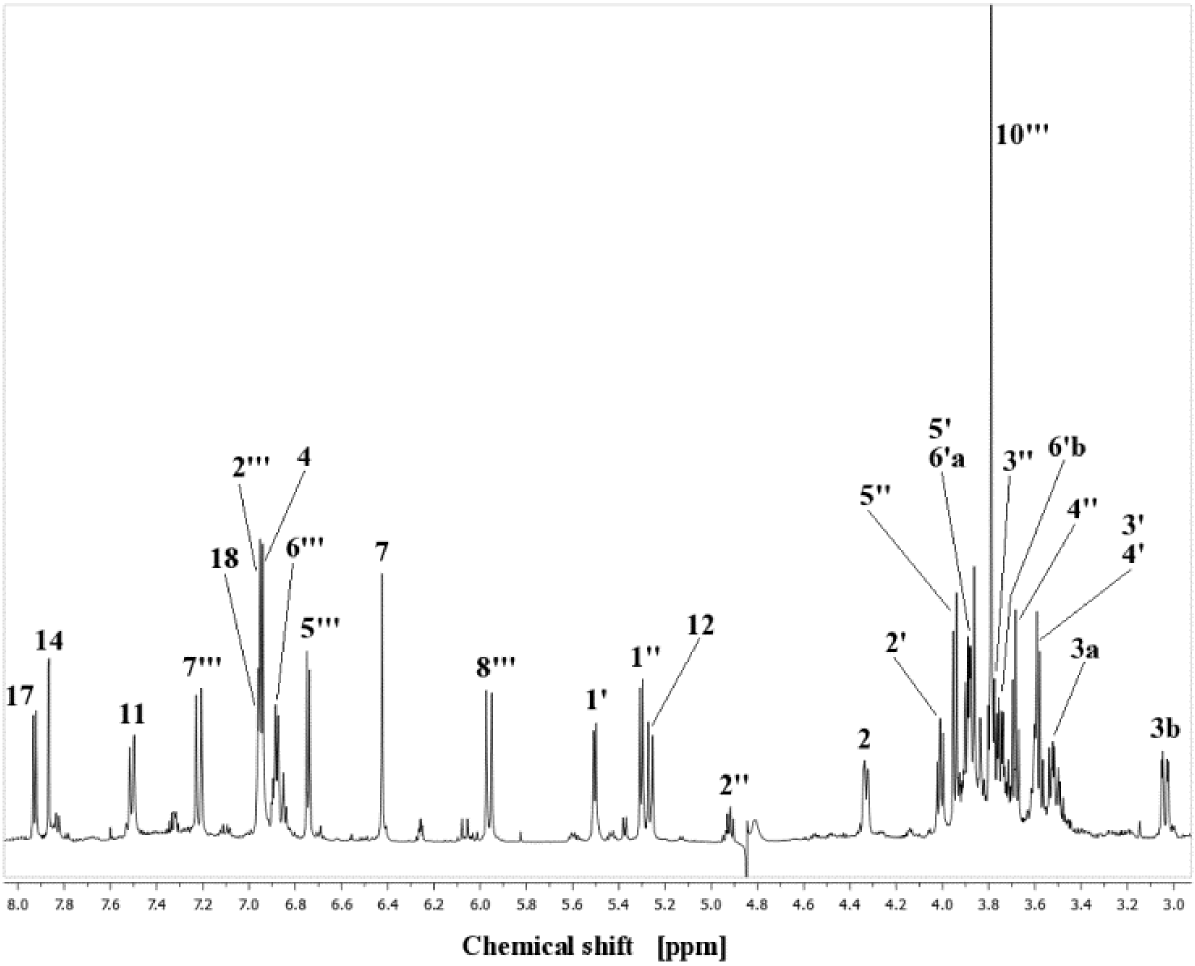

Figure S2 ^{1}H NMR spectrum of 17-decarboxy-neocelosianin (D_2O, 295 K).

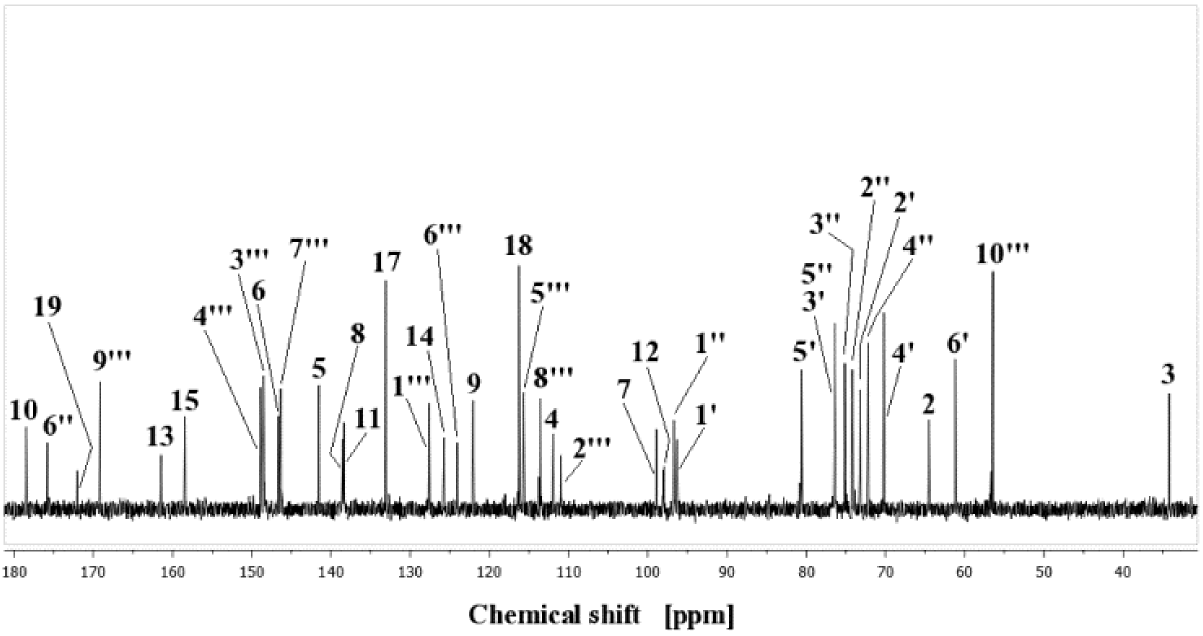

Figure S3 ^{13}C NMR spectrum of 17-decarboxy-neocelosianin (D_2O, 295 K).

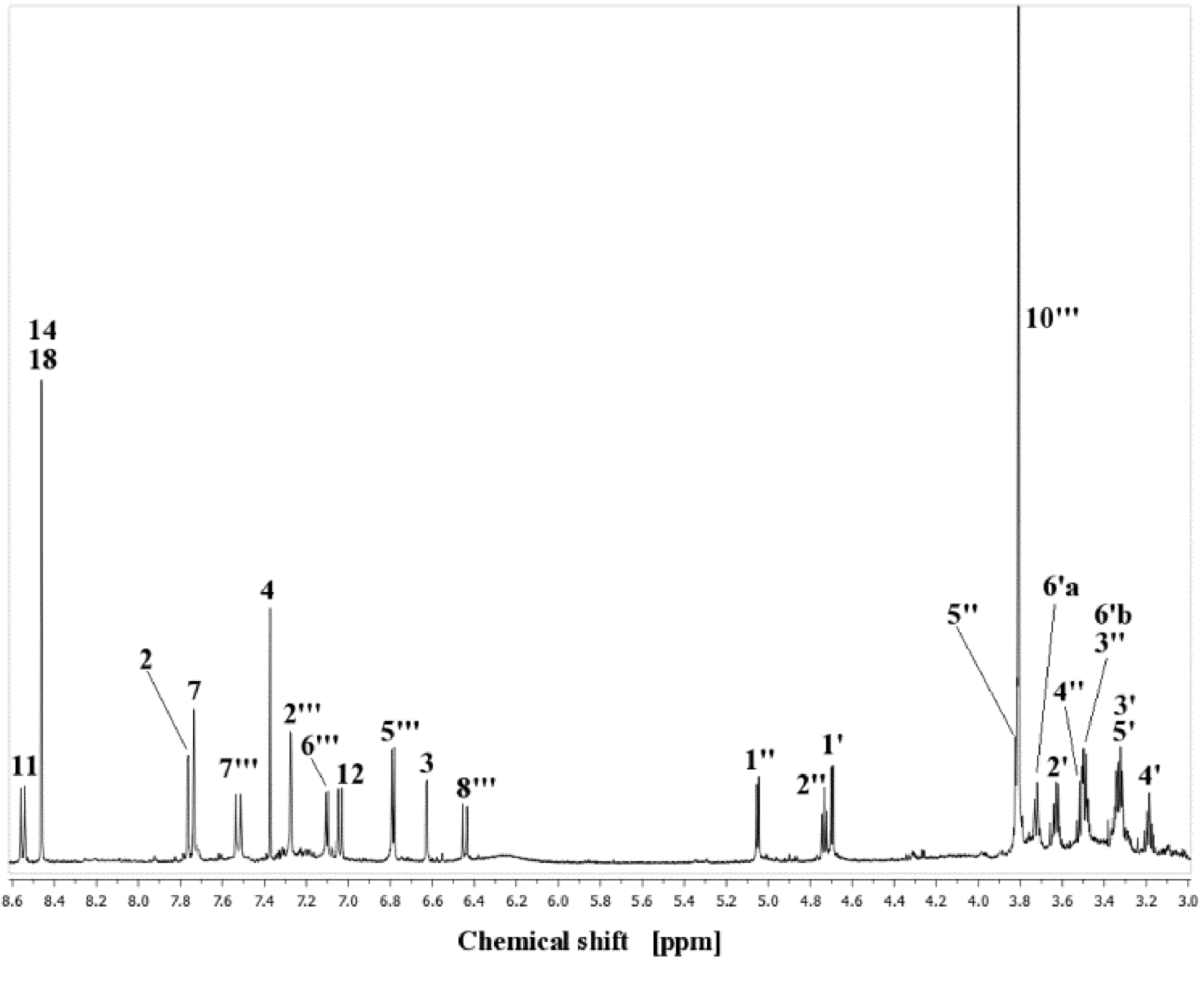

Figure S4 ^{1}H NMR spectrum of 2-decarboxy-xanneocelosianin (d6-DMSO/d-TFA, 295 K).

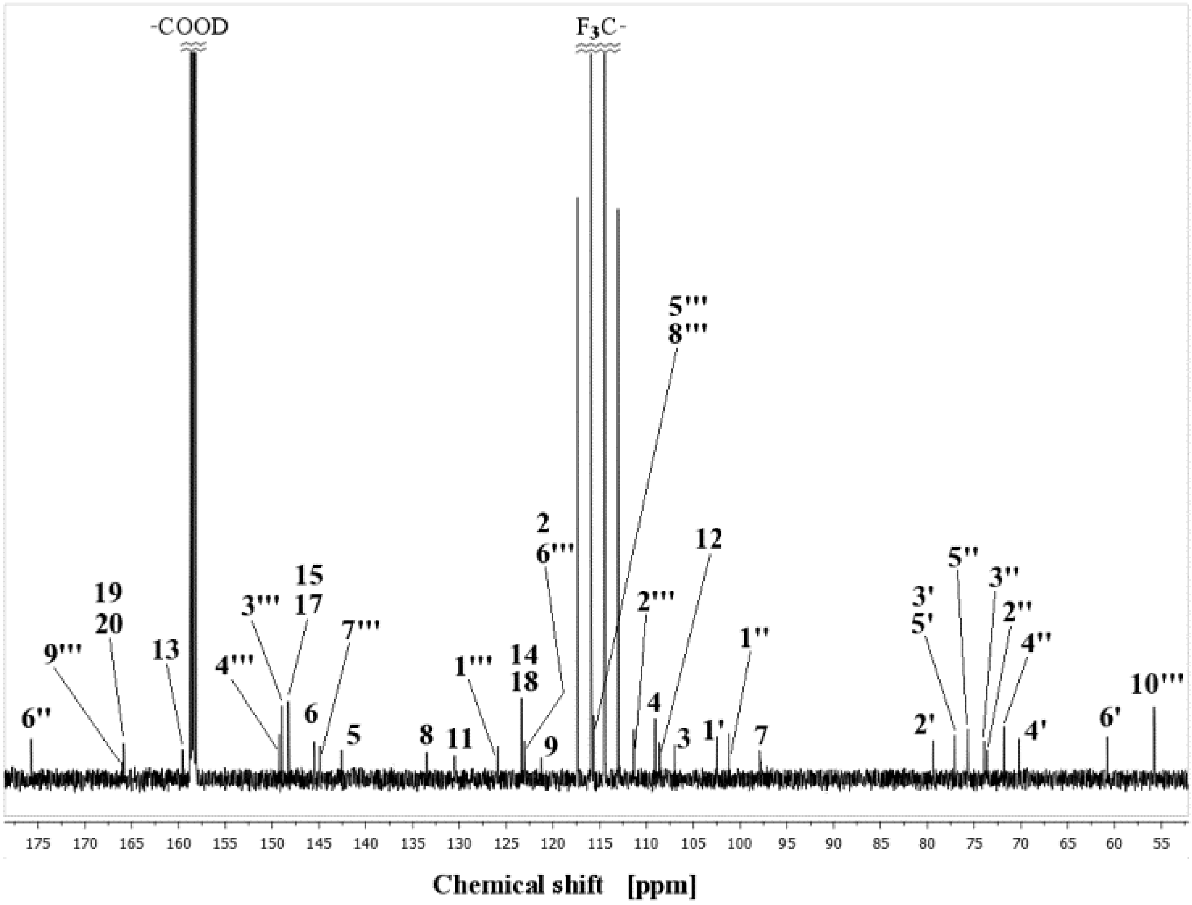

Figure S5 ^{13}C NMR spectrum of 2-decarboxy-xanneocelosianin (d6-DMSO/d-TFA, 295 K).

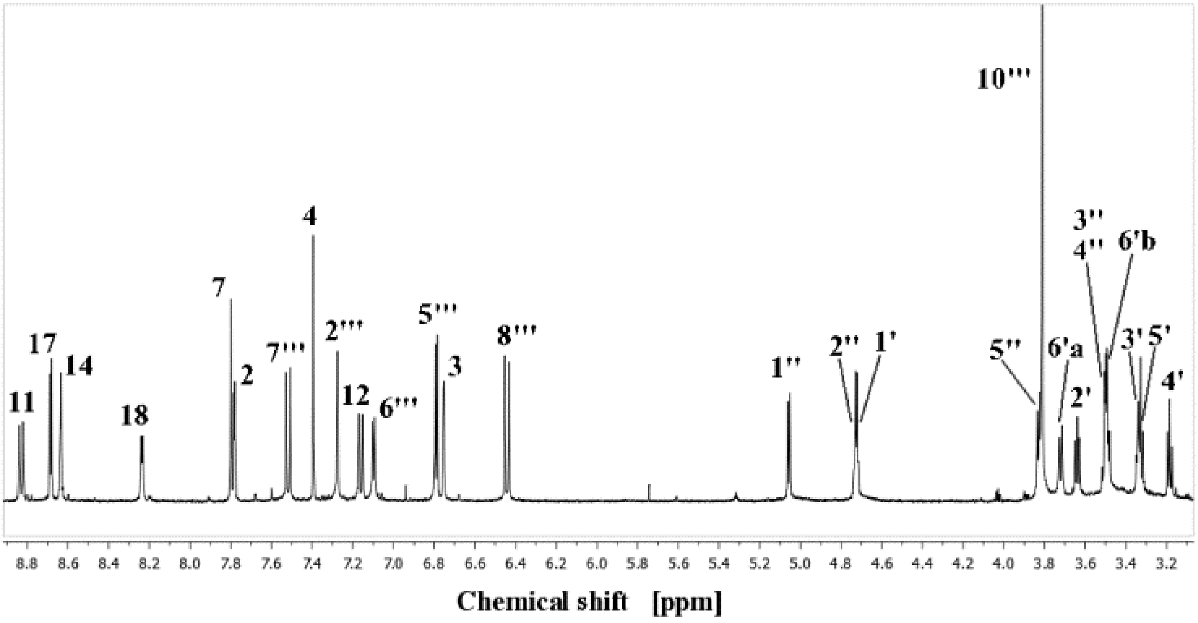

Figure S6 ^{1}H NMR spectrum of 2,17-bidecarboxy-xanneocelosianin (*d6*-DMSO/*d*-TFA, 295 K).

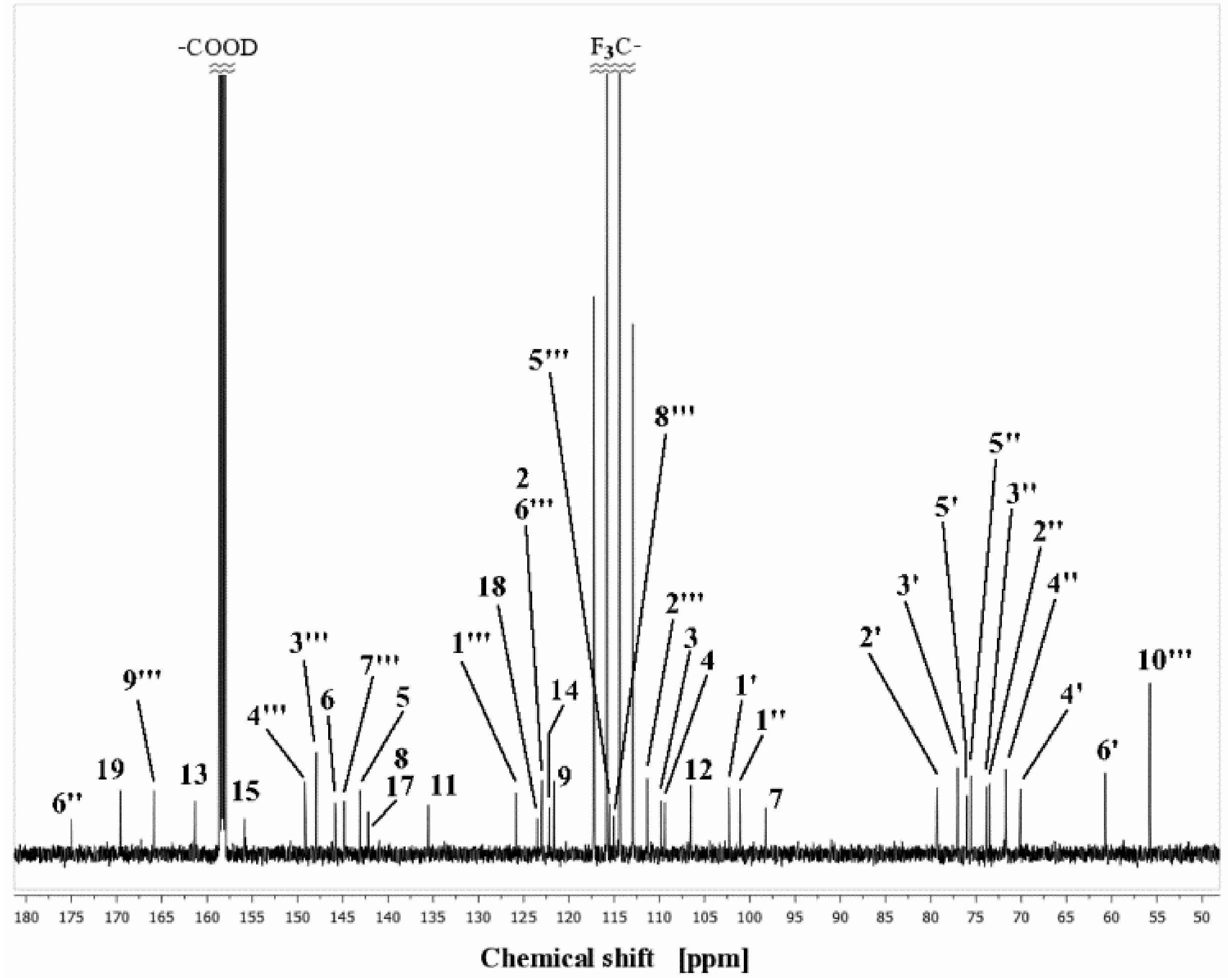

Figure S7 ^{13}C NMR spectrum of 2,17-bidecarboxy-xanneocelosianin (d6-DMSO/d-TFA, 295 K).

Antioxidant activity of oxidized celosianins compared to the initial pigment and reference compound gallic acid

The antioxidant activity of the tested compounds was evaluated using the ABTS, FRAP, and ORAC assays. The ABTS assay results were expressed as the IC_{50} value (mg/L) (Figure S8, Table S2), and the results for all assays were further expressed as milimoles ofTrolox per gram of dry weight of the sample (mmol Trolox/g DW in all assays) (Table S2).

Table S2 Antioxidant activity of celosianin and its oxidation products by means of the ABTS, FRAP and ORAC assays. The results are expressed as IC_{50} in μg/mL and TEAC values in mmol Trolox/g DW.

Sample	IC_{50}	$TEAC_{ABTS}$	$TEAC_{FRAP}$	$TEAC_{ORAC}$
	mg/L		mmol Trolox/g DW	
Cel	$23^{c} \pm 1.2$	$2.4^{b} \pm 0.062$	$2.0^{b} \pm 0.10$	$8.1^{b} \pm 0.15$
17-dNCel	$34^{a} \pm 1.4$	$1.8^{c} \pm 0.086$	$1.0^{d} \pm 0.051$	$5.9^{c} \pm 0.26$
2,17-dXNCel	$29^{b} \pm 1.3$	$2.0^{c} \pm 0.077$	$1.2^{c} \pm 0.058$	$8.0^{b} \pm 0.096$
GA	$5.2^{d} \pm 0.33$	$7.5^{a} \pm 0.093$	$5.5^{a} \pm 0.022$	$11.4^{a} \pm 0.18$

Mean ± standard deviation (n=3). Different letters in columns indicate statistically significant differences ($p \leq 0.05$) between samples according to Tuckey's test

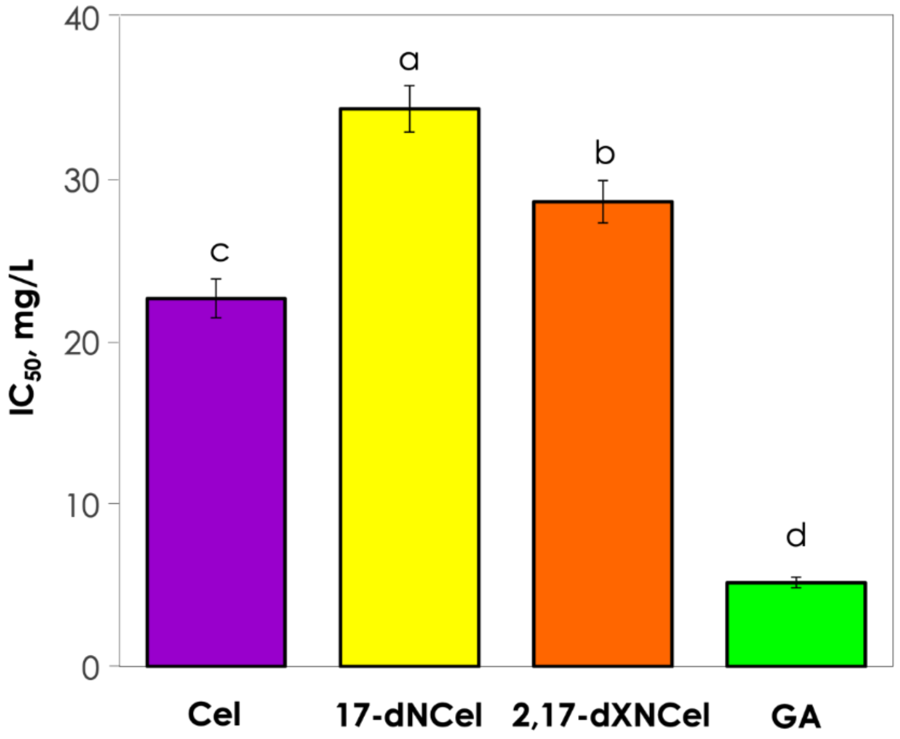

Figure S8 IC_{50} values of celosianin **17**, and its oxidation products (17-decarboxy-neocelosianin **18**, and 2,17-bidecarboxy-xanneocelosianin **20**) as well as standard gallic acid calculated based on ABTS assay.

Determination of H9c2 cells morphology

The cell morphology of rat cardiac myoblasts (H9c2), pretreated with various concentrations of celosianin and its oxidation products, was assessed based on fluorescent live/dead imaging. The cells were imaged using a Zeiss Axiovert 40 fluorescence microscope (magnification 100x) equipped with a HXP 120C metal halide illuminator (Carl Zeiss, Germany).

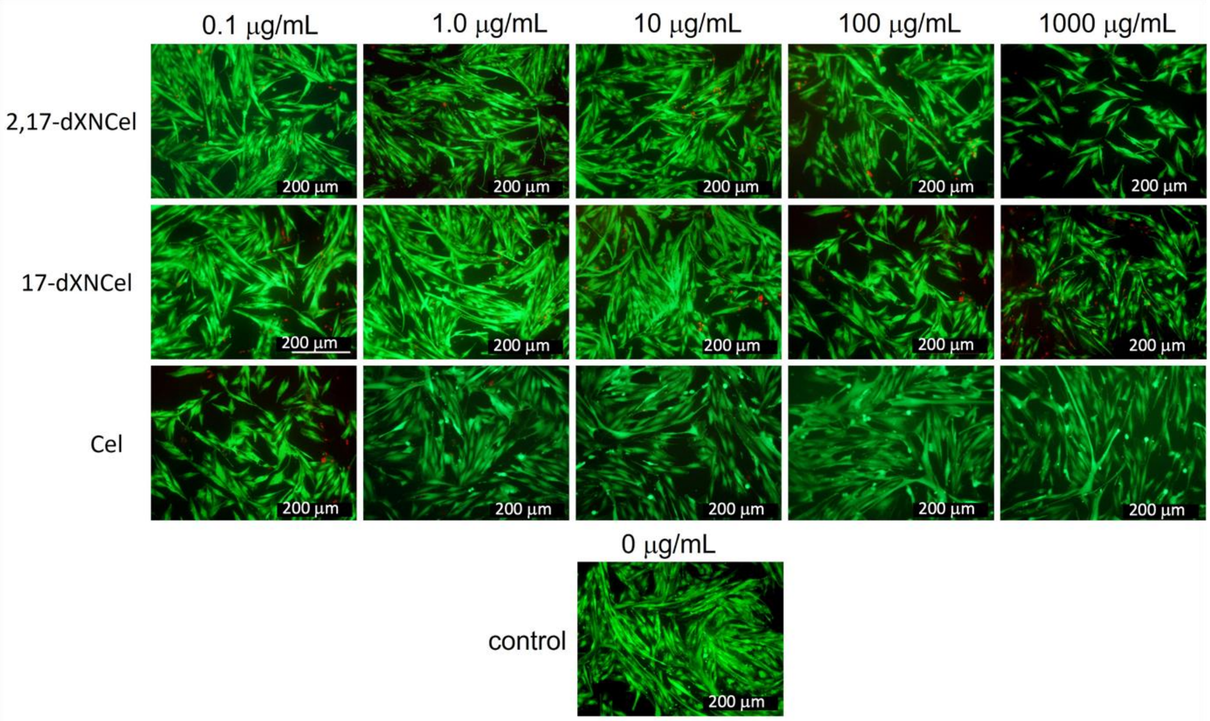

Figure S9 Live/dead staining of H9c2 cells incubated in the presence of 2,17-dXNCel, 17-dNCel, Cel and for control without pigments. Scale bare 200 µm.
